# The Endoplasmic Reticulum-Mitochondrion Tether ERMES Orchestrates Fungal Immune Evasion, Illuminating Inflammasome Responses to Hyphal Signals

**DOI:** 10.1128/mSphere.00074-16

**Published:** 2016-05-25

**Authors:** Timothy M. Tucey, Jiyoti Verma-Gaur, Julie Nguyen, Victoria L. Hewitt, Tricia L. Lo, Miguel Shingu-Vazquez, Avril A. B. Robertson, James R. Hill, Filomena A. Pettolino, Travis Beddoe, Matthew A. Cooper, Thomas Naderer, Ana Traven

**Affiliations:** a Infection and Immunity Program and the Department of Biochemistry and Molecular Biology, Biomedicine Discovery Institute, Monash University, Clayton, Victoria, Australia; b Institute for Molecular Bioscience, University of Queensland, Brisbane, Australia; c CSIRO Agriculture, Canberra, Australian Capital Territory, Australia; d Department of Animal, Plant and Soil Science, AgriBio Centre for AgriBioscience, La Trobe University, Melbourne (Bundoora), Victoria, Australia; Carnegie Mellon University

**Keywords:** *Candida albicans*, macrophage, metabolism, mitochondria

## Abstract

The pathogenic yeast *Candida albicans* escapes macrophages by triggering NLRP3 inflammasome-dependent host cell death (pyroptosis). Pyroptosis is inflammatory and must be tightly regulated by host and microbe, but the mechanism is incompletely defined. We characterized the *C. albicans* endoplasmic reticulum (ER)-mitochondrion tether ERMES and show that the ERMES *mmm1* mutant is severely crippled in killing macrophages despite hyphal formation and normal phagocytosis and survival. To understand dynamic inflammasome responses to *Candida* with high spatiotemporal resolution, we established live-cell imaging for parallel detection of inflammasome activation and pyroptosis at the single-cell level. This showed that the inflammasome response to *mmm1* mutant hyphae is delayed by 10 h, after which an exacerbated activation occurs. The NLRP3 inhibitor MCC950 inhibited inflammasome activation and pyroptosis by *C. albicans*, including exacerbated inflammasome activation by the *mmm1* mutant. At the cell biology level, inactivation of ERMES led to a rapid collapse of mitochondrial tubular morphology, slow growth and hyphal elongation at host temperature, and reduced exposed 1,3-β-glucan in hyphal populations. Our data suggest that inflammasome activation by *C. albicans* requires a signal threshold dependent on hyphal elongation and cell wall remodeling, which could fine-tune the response relative to the level of danger posed by *C. albicans*. The phenotypes of the ERMES mutant and the lack of conservation in animals suggest that ERMES is a promising antifungal drug target. Our data further indicate that NLRP3 inhibition by MCC950 could modulate *C. albicans*-induced inflammation.

**IMPORTANCE** The yeast *Candida albicans* causes human infections that have mortality rates approaching 50%. The key to developing improved therapeutics is to understand the host-pathogen interface. A critical interaction is that with macrophages: intracellular *Candida* triggers the NLRP3/caspase-1 inflammasome for escape through lytic host cell death, but this also activates antifungal responses. To better understand how the inflammasome response to *Candida* is fine-tuned, we established live-cell imaging of inflammasome activation at single-cell resolution, coupled with analysis of the fungal ERMES complex, a mitochondrial regulator that lacks human homologs. We show that ERMES mediates *Candida* escape via inflammasome-dependent processes, and our data suggest that inflammasome activation is controlled by the level of hyphal growth and exposure of cell wall components as a proxy for severity of danger. Our study provides the most detailed dynamic analysis of inflammasome responses to a fungal pathogen so far and establishes promising pathogen- and host-derived therapeutic strategies.

## Abstract (precis)

The yeast *Candida albicans* causes human infections that have mortality rates approaching 50%. The key to developing improved therapeutics is to understand the host-pathogen interface. A critical interaction is that with macrophages: intracellular *Candida* triggers the NLRP3/caspase-1 inflammasome for escape through lytic host cell death, but this also activates antifungal responses. To better understand how the inflammasome response to *Candida* is fine-tuned, we established live-cell imaging of inflammasome activation at single-cell resolution, coupled with analysis of the fungal ERMES complex, a mitochondrial regulator that lacks human homologs. We show that ERMES mediates *Candida* escape via inflammasome-dependent processes, and our data suggest that inflammasome activation is controlled by the level of hyphal growth and exposure of cell wall components as a proxy for severity of danger. Our study provides the most detailed dynamic analysis of inflammasome responses to a fungal pathogen so far and establishes promising pathogen- and host-derived therapeutic strategies.

## INTRODUCTION

Important aspects of microbial pathogenesis involve metabolic adaptation in the host (1–3). The yeast *Candida albicans* is the most common fungal pathogen in human infections, and it can cause deadly systemic disease (4). For *C. albicans*, metabolic regulation has wide-ranging consequences for virulence, including interaction with host immunity and resistance to stressors and antifungal therapeutics (reviewed in reference 3). In eukaryotic cells, mitochondria have central functions in energy production and metabolism, and mitochondrial function is necessary for virulence of pathogenic fungi (reviewed in references 5 and 6). Therefore, the development of new antifungal strategies based on metabolic and mitochondrial regulation is promising, but few regulators of these pathways have been characterized in fungal pathogens.

Metabolic adaptation is important for *C. albicans* during the critical immune interaction with macrophages. *C. albicans* reprograms its metabolism to suit the nutrient environment in phagocytes (7, 8). Furthermore, metabolism is involved in the transition of *C. albicans* from yeast to hyphal morphology, which promotes immune evasion by causing host cell lysis (9–12). Recent work from our lab and the Krysan lab has shown that, upon phagocytosis, hyphae rapidly trigger a programmed macrophage cell death mechanism termed pyroptosis (13, 14). Pyroptosis exposes intracellular pathogens to immune attack and rids them of their replication niche (15). Given that *C. albicans* primarily replicates extracellularly, we have proposed that this fungus “hijacks” pyroptosis to egress and evade intracellular killing (13). Induction of pyroptosis by *C. albicans* depends on the NLRP3/caspase-1 inflammasome (13, 14). Activation of the NLRP3 inflammasome by *Candida* needs to be tightly regulated by both pathogen and host. On the pathogen side, filamentous growth has been linked to inflammasome activation and pyroptosis (13, 14, 16). This suggests that *C. albicans* induces pyroptosis after the expression of virulence traits that may be important for extracellular survival and dissemination under inflammatory conditions, as activation of the NLRP3 inflammasome also triggers antifungal immune responses (reviewed in references 17 and 18). How inflammasome activation by *C. albicans* is tightly regulated remains to be fully understood, particularly in light of recent studies that showed that factors other than fungal morphology are at play, and yeast cells can also cause inflammasome-dependent macrophage lysis under some conditions (13, 14, 19). On the host side, inflammasome activation by *C. albicans* needs to be regulated to modulate inflammation in response to commensal or pathogenic fungal growth. Besides pyroptosis, *C. albicans* triggers other, less-defined forms of macrophage death, most strikingly a second wave of killing that eventually eliminates the entire macrophage population (13, 14). We termed these two stages of killing phase 1 (pyroptotic death) and phase 2 (nonpyroptotic death) (13). Fungal factors coordinating these distinct macrophage death pathways are unknown.

Here, we sought to characterize novel regulators that mediate evasion of macrophages by *Candida*, focusing on fungal mitochondria, which have so far been largely understudied in this context. For this, we characterized a key mitochondrial regulator, the endoplasmic reticulum (ER)-mitochondrion tethering complex ERMES (20). Complexes such as ERMES, which mediate interactions between organelles by providing “membrane contact sites,” represent hubs that can control cell physiology on a global level (21, 22). The functions of such complexes are poorly understood in eukaryotic pathogens. ERMES is particularly promising in the context of fungal pathogenesis because it is found broadly in fungi but is absent from animals (23), and it could therefore be targeted for antifungal therapy. In support of ERMES being a promising antifungal drug target, a mutant library screen by Merck identified the ERMES subunit *MMM1* as being important for *C. albicans* virulence in the mouse tail vein infection model of candidiasis (24). However, the cellular functions of ERMES in *C. albicans* and its potential roles in host-pathogen interactions have not been studied so far.

We report here that in *C. albicans* the activity of ERMES is important for enabling immune evasion via multiple macrophage death mechanisms (phase 1 and phase 2). Key roles of ERMES in *C. albicans* are the regulation of mitochondrial morphology and enabling optimal growth at host temperature. To further understand the interplay between *C. albicans* and the inflammasome, we established live-cell imaging to monitor inflammasome activation and macrophage death in parallel, at single-cell resolution and in real time over the entire interaction course of 24 h (i.e., until the entire macrophage culture collapses). We combined this powerful assay with ERMES mutant analysis and a newly described small-molecule inhibitor of NLRP3 that showed promise in treating inflammatory disorders (25). Using these novel tools, we show that the inflammasome not only responds to fungal morphotype but also discriminates hyphae produced by wild-type *C. albicans* from hyphae produced by the less virulent ERMES mutant. We propose that this discrimination is achieved through a signal threshold response for inflammasome activation that is linked to hyphal growth and cell wall remodeling. Based on our data, we suggest novel host- and pathogen-derived avenues for antifungal drug development and propose that our results and the imaging assay that we established will be broadly applicable to the understanding of dynamic inflammasome responses to fungal pathogens.

## RESULTS

### *C. albicans* ERMES is required for macrophage killing and immune evasion.

**FIG 1:**
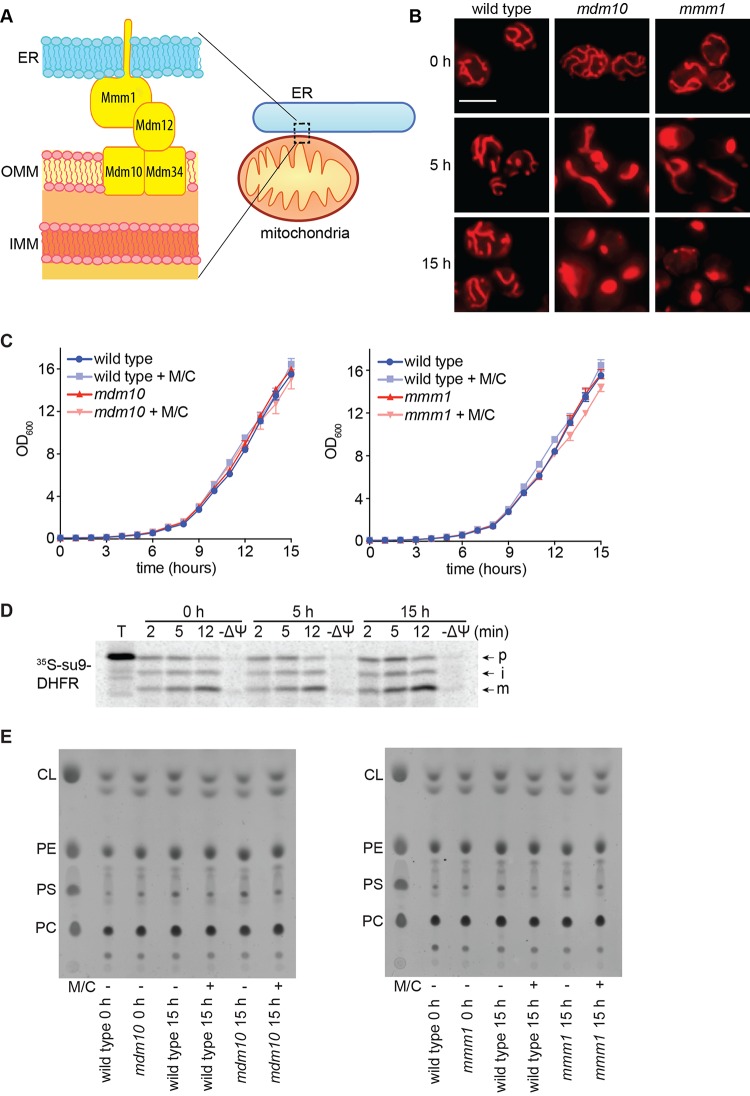
The *Candida* ERMES has a primary role in mitochondrial morphology. (A) Cartoon of the core ERMES complex as understood in *S. cerevisiae*. Recent work has shown that Mmm1 and Mdm12 associate as a heterotetramer and that Mdm34 also forms dimers (58), but for simplicity, we do not depict it here. The precise architecture of the entire complex is yet to be understood. ER, endoplasmic reticulum; OMM, outer mitochondrial membrane; IMM, inner mitochondrial membrane. (B) Loss of mitochondrial morphology upon ERMES inactivation monitored at 30°C. Shown are images of representative cells selected from larger microscopy fields depicted in Fig. S2 in the supplemental material. Bar, 5 µm. Quantification is in Fig. S3 in the supplemental material. (C) Growth curves of the indicated strains under permissive or repressive (+ M/C) conditions at 30°C. Shown are averages and the standard errors of the means from 3 biological replicates assayed in the same experiment. OD_600_, optical density at 600 nm. (D) Mitochondria were prepared from the *mdm10* strain before or at 5 h and 15 h after gene repression and incubated with ^35^S-labeled mitochondrial reporter Su9-dihydrofolate reductase (DHFR) for the indicated times. Mitochondrial membrane potential was dissipated before import in the −ΔΨ lanes. Mitochondria were analyzed by SDS-PAGE and phosphorimaging. p, precursor; i, intermediate; m, mature processed form. (E) Total cellular lipids were extracted following growth under permissive or repressive (+ M/C) conditions for 15 h and separated by thin-layer chromatography. Only phospholipids are shown here: CL, cardiolipin; PE, phosphatidylethanolamine; PC, phosphatidylcholine; PS, phosphatidylserine. Quantification is in Fig. S4 in the supplemental material.

In *C. albicans*, mitochondrial dysfunction can have large effects on fitness (for example, see reference 26). Therefore, to start delineating the functions of ERMES, we constructed conditional mutants in two ERMES genes: *MMM1*, which encodes the subunit anchored in the ER, and *MDM10*, which encodes a subunit located in the mitochondrial outer membrane (Fig. 1A). In these mutants, one allele is deleted and the other one is placed under the *MET3* promoter, which is “on” in the absence of methionine and cysteine and “off” in their presence. Gene repression was achieved following addition of methionine and cysteine to the medium (see Fig. S1A in the supplemental material), and microscopy showed that under these conditions ERMES function is inactivated, as both mutants displayed an early and clear defect in mitochondrial morphology (Fig. 1B; see also Fig. S2). Already at 5 h postrepression, loss of mitochondrial tubular network structure was observed, and the defect was even more pronounced after 15 h, with the clear appearance of globular, collapsed mitochondria (Fig. 1B; see also quantification in Fig. S3). This mitochondrial morphology defect is consistent with what is observed in ERMES mutants of *Saccharomyces cerevisiae* (27–30). Unlike mitochondrial morphology, the growth of the two mutants was not compromised to a considerable degree even after several cell divisions in the 15-h time course (Fig. 1C). Consistent with normal respiration, mitochondria isolated from the *mdm10* mutant maintained their membrane potential upon repression, as they imported a substrate normally into the mitochondrial matrix (Fig. 1D). Moreover, steady-state levels of cellular phospholipids in both mutants were the same as those in the wild type at 15 h postrepression, and this included the mitochondrion-specific cardiolipin (Fig. 1E; see quantification in Fig. S4). Studies in *S. cerevisiae* suggested roles for ERMES not only in mitochondrial morphology but also in mitochondrial lipid homeostasis, fitness, and respiration through maintenance of the mitochondrial genome (20, 27–34). To further address these additional functions of ERMES in *C. albicans*, we made homozygous deletion mutants in each one of the four ERMES subunits. The four mutants had equivalent phenotypes and displayed large fitness defects with barely viable cells, lack of growth on glycerol, lack of a wild-type mitochondrial network, and altered lipid homeostasis through loss of the mitochondrial phospholipid cardiolipin (see Fig. S5). Collectively, our results show that in *C. albicans* the earliest defect upon ERMES inactivation is loss of wild-type mitochondrial morphology, while the lipid, respiration, and fitness defects are seen following longer-term inactivation of ERMES. While our data show that, of all the mutant phenotypes tested, mitochondrial morphology is the most sensitive to ERMES gene repression, it is possible that residual ERMES protein levels are present in the conditional mutants, and this could support functions in mitochondrial lipid homeostasis and fitness.

**FIG 2:**
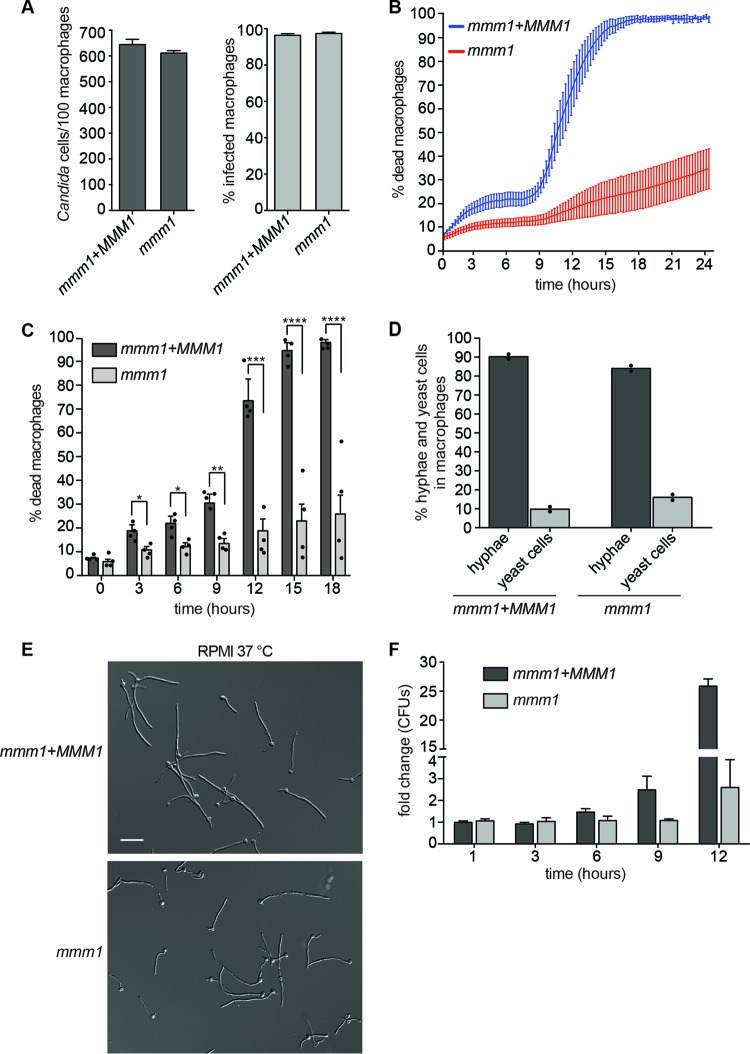
ERMES is required for macrophage killing and fungal escape. (A) The phagocytic index (number of *Candida* cells per 100 macrophages) and the percentage of infected macrophages were determined at 1 h postphagocytosis. The MOI was 6 *Candida* cells to 1 macrophage. Shown are averages and the standard errors of the means from 3 independent experiments. (B) Macrophage cell death over time. Time zero is the start of live-cell imaging, after coincubation of *C. albicans* with macrophages for 1 h and washing of nonphagocytosed cells. Shown are averages from 4 independent experiments and the standard errors. See also Movies S1 and S2 in the supplemental material. (C) Bar graphs of selected time points from panel B, with each experimental data point shown in the scatter plot overlay. Statistical significance was determined by unpaired *t* test with Welch’s correction. *, *P* < 0.05; **, *P* < 0.01; ***, *P* < 0.001; ****, *P* < 0.0001. (D) Quantification of hyphal formation in macrophages after 1 h of coincubation and washes. Data are from 2 independent experiments (shown separately as dot points and the mean), and 200 cells were measured for each strain per experiment. “Hyphae” represent germ tubes plus hyphal filaments. (E) Hyphal formation in repressive RPMI medium, 3 h at 37°C. Bar, 20 µm. (F) Fungal CFU were determined at the indicated time points following phagocytosis. After macrophage lysis, fungal cells were plated onto medium permissive for growth of the *mmm1* mutant. Shown are the averages and standard errors of the means from 3 independent experiments. CFU fold change was calculated by normalizing to the control strain at 1 h.

10.1128/mSphere.00074-16.2 Figures S1 to S7 (Fig. S1) Analysis of ERMES gene repression. (A) Cells of the indicated strains were grown under permissive conditions (synthetic medium without methionine and cysteine) followed by addition of 2.5 mM methionine and 0.5 mM cysteine at time zero, and gene repression was monitored by quantitative RT-PCR at the indicated time points. The levels of ERMES genes were normalized to the levels of the RNA polymerase III transcript *SCR1* or the rRNA transcript *RDN5* or *RDN25*. Shown are averages and standard errors from 3 biological replicates assayed in the same experiment. (B) *MMM1* gene repression in macrophages. The *mmm1* mutant and the complemented control strain were grown at 30°C under repressive conditions overnight. Macrophages were infected with *C. albicans* at the multiplicity of infection of 6 *Candida* cells to 1 macrophage, with methionine and cysteine included in the tissue culture medium. Gene repression was monitored by quantitative RT-PCR at the indicated time points following coincubation of *C. albicans* with macrophages and washing of the nonphagocytosed cells. To separate *Candida* from the macrophages, Trizol treatment was performed to remove lysed macrophages, and subsequently, *Candida* cell pellets were recovered and RNA was isolated by the hot phenol method. Transcript levels of *MMM1* were normalized to the RNA polymerase III transcript *SCR1* or the rRNA transcript *RDN5*. Shown are averages and the standard errors from 4 biological replicates obtained from 2 independent experiments.
(Fig. S2) Mitochondrial morphology defects of ERMES mutant strains. Shown are larger microscopy fields of the MitoTracker Red images displayed in Fig. 1B, without any processing (i.e., color or contrast correction) of the images. Bar, 10 µm.
(Fig. S3) Quantification of the mitochondrial morphology defects after conditional inactivation of ERMES. Relates to Fig. 1B. Percentages of cells with abnormal mitochondria in wild-type and *mdm10* (top) and *mmm1* (bottom) strains are shown. This was calculated at 0, 5, and 15 h postrepression from 3 independent biological replicates for each of the strains (3 independent colonies) assayed in the same experiment, counting at least 300 cells for each repeat. In parallel, cells were grown under permissive conditions as a control. Impaired mitochondria were quantified based on any mitochondria not resembling the wild-type tubular network as detected by MitoTracker Red staining, and this was subdivided into two distinct categories: a milder phenotype (“shrunken”), where the tubular network was reduced in size and not spanning the entire cell when analyzing multiple planes of focus under the microscope, and a severe phenotype (“collapsed”), consisting of round, enlarged, or severely fragmented mitochondria. The experiment was performed on multiple occasions with equivalent results observed.
(Fig. S4) Quantification of phospholipids after conditional inactivation of ERMES. Relates to Fig. 1E. Lipids were quantified using the Toolbox module of ImageQuant 1D version 7.0, and background signals were subtracted using the local median method performed by the software. Shown are averages and the standard deviations from three biological repeats. The amounts of cardiolipin (CL) and phosphatidylethanolamine (PE) are shown relative to the levels of phosphatidylcholine (PC) in the same sample as an internal control and in comparison to the wild type.
(Fig. S5) Long-term inactivation of ERMES results in loss of fitness, altered mitochondrial network morphology, and phospholipid imbalance. (A) (Left) Wild-type *C. albicans* and homozygous deletion strains lacking *MDM12* (*mdm12ΔΔ*), *MMM1* (*mmm1ΔΔ*), *MDM10* (*mdm10ΔΔ*), or *MDM34* (*mdm34ΔΔ*) were grown on rich medium containing glucose (YPD) or glycerol (YPG) as a carbon source, at 30°C or 37°C. Tenfold serial dilutions were plated, starting from an optical density at 600 nm (OD_600_) of 0.5. Images were taken after 3 days. (Right) Complementation of the fitness defects of the homozygous deletion strains for *MDM12* (*mdm12ΔΔ+12*), *MMM1* (*mmm1ΔΔ+1*), and *MDM34* (*mdm34ΔΔ+34*). (B) Representative fluorescence images of the wild-type and ERMES homozygous mutant strains stained with MitoTracker Green dye. Bar, 5 µm. Images grouped together were all taken in parallel in the same experiment. No wild-type mitochondrial network morphology was seen in the homozygous ERMES mutants. Complementation of the mitochondrial network defects is shown for *MDM12* (*mdm12ΔΔ+12*), *MMM1* (*mmm1ΔΔ+1*), and *MDM34* (*mdm34ΔΔ+34*). We noticed that in complemented strains with expression levels of the ERMES genes equivalent to approximately half of wild-type levels (as expected from single-copy gene reintegrants), restoration of mitochondrial morphology defects was partial; clones with expression levels equivalent to 2- to 3-fold over wild-type values displayed significantly better complementation of mitochondrial morphology. Elevated levels of ERMES genes may be necessary for restoring growth to severely sick ERMES mutants. (C) Total cellular lipids were extracted from the indicated strains and separated by thin-layer chromatography (TLC). Standards were used to identify cardiolipin (CL), phosphatidylethanolamine (PE), phosphatidylserine (PS), and phosphatidylcholine (PC). The right panel displays quantification averages, performed using ImageQuant, from 3 biological repeats. Error bars indicate standard deviations. ***, *P* < 0.0005; ****, *P* < 0.0001, compared to wild type using Student’s *t* test (unpaired, two-tailed). The amounts of CL and PE are shown relative to the levels of PC in the same sample as an internal control and in comparison to the wild type.
(Fig. S6) Effects of methionine and cysteine addition in the macrophage-interaction experiments. Relates to Fig. 2B. Macrophages were infected with the *mmm1* mutant and the complemented control strain at an MOI of 6:1 (*Candida* cells to macrophage). After 1 h of coincubation and washing of nonphagocytosed cells, macrophage medium was included with (+Met/Cys) or without (-Met/Cys) 2.5 mM methionine and 0.5 mM cysteine. Live-cell imaging was then started 3 h later. Shown are averages and standard errors from 2 biological replicates (2 independent colonies of each of the two *C. albicans* strains) assayed in the same experiment.
(Fig. S7) Characterization of hyphal growth of the *mmm1* mutant. (A) Relates to Fig. 2E. Mitochondrial network structure of hyphae was visualized by staining with MitoTracker Red dye. Representative images of several hyphae made by the *mmm1* mutant and the complemented control strain are shown in the left panel. The right panel contains a higher magnification of a single hyphal cell with bright-field overlay. In both images, the scale bar represents 10 µm. (B) Relates to Fig. 5C. Hyphal length distribution in RPMI medium after 3 h of growth. Germ tubes or hyphal filaments were over 5 µm. Each cell measured is depicted by a line, with length shown on the *y* axis and the measurements ranked in order from smallest to largest on the *x* axis. Three biological replicates were performed. Two repeats are shown here, and the third repeat is shown in Fig. 5C. (C) Relates to Fig. 5E. Hyphal length distribution in macrophages following 1 h of coincubation and washes was measured in 2 independent experiments with equivalent results (*n* = 200 fungal cells per strain). One experiment is shown here, and the other one is shown in Fig. 5E. Download Figures S1 to S7, PDF file, 8.5 MB. Copyright © 2016 Tucey et al. 2016 Tucey et al. This content is distributed under the terms of the Creative Commons Attribution 4.0 International license.

Next, we tested how ERMES might be important for *Candida*-macrophage interactions. The experiments were done under repressive conditions (in the presence of methionine and cysteine), using the conditional *mmm1* mutant and the complemented *mmm1*+*MMM1* strain as the control. Quantitative reverse transcription-PCR (RT-PCR) analysis showed that repression of the *MMM1* gene was maintained in macrophages for at least 15 h (see Fig. S1B in the supplemental material). Addition of methionine and cysteine to the medium did not change the progression of macrophage killing by the control *C. albicans* strain (see Fig. S6). Moreover, the macrophage killing curve obtained under these conditions was comparable to our previous results in medium without additional supplementation with methionine and cysteine (13). The *mmm1* mutant was phagocytosed normally by bone marrow-derived macrophages (BMDMs) (Fig. 2A), but live-cell imaging showed that its ability to kill macrophages was severely compromised, with macrophage death reaching only ≈30% by 24 h (Fig. 2B and C; see also Movies S1 and S2). As a control, we show that derepression of the *MMM1* gene by omission of methionine and cysteine from the medium during the *C. albicans*-macrophage interaction experiment resulted in higher macrophage killing by the ERMES mutant than that obtained under repressive conditions (see Fig. S6). Despite highly compromised host cell killing, the *mmm1* mutant was able to undergo hyphal morphogenesis in macrophages (Fig. 2D). Hyphal formation by the mutant was also evident in macrophage growth medium *in vitro* (Fig. 2E; see also Fig. S7A), and the mutant hyphae continued to grow over the course of the macrophage experiment (see Movie S2). The *mmm1* mutant maintained viability in macrophages for at least 12 h postinfection (Fig. 2F). In the first 6 h postinfection, *Candida* CFU derived from infected macrophages were similar between the *mmm1* mutant and the control strain (Fig. 2F). At 9 h postinfection, some increase in CFU was seen for the control strain, and at 12 h postinfection, control strain CFU clearly increased, while the increase in mutant CFU was diminished (Fig. 2F). This is consistent with substantial escape of the control *Candida* strain from macrophages, as most fungal growth under these conditions is seen after hyphae lyse macrophages and egress into the surrounding medium (see Movie S1). In this study and previously, we observed that after escape from macrophages into extracellular medium, growth of yeast-form cells coincides with phase 2, pyroptosis-independent macrophage death (see Movie S1) (13). Unlike in control samples, in infections with the *mmm1* mutant no substantial yeast growth was observed in the medium at later time points (compare Movies S1 and S2 in the supplemental material).

10.1128/mSphere.00074-16.4 Movie S1  Murine bone marrow-derived macrophages (BMDMs) infected with the control *C. albicans* strain (*mmm1+MMM1*). To create the video files, using Fiji software, the TIFF hyperstack was exported as an AVI file with JPEG compression at 3.0 frames per s. For all data analysis, the images were kept in TIFF format. Download Movie S1, AVI file, 14.2 MB. Copyright © 2016 Tucey et al. 2016 Tucey et al. This content is distributed under the terms of the Creative Commons Attribution 4.0 International license.

10.1128/mSphere.00074-16.5 Movie S2  Murine bone marrow-derived macrophages (BMDMs) infected with the *mmm1* mutant strain. The file was created as described for Movie S1. Download Movie S2, AVI file, 11 MB. Copyright © 2016 Tucey et al. 2016 Tucey et al. This content is distributed under the terms of the Creative Commons Attribution 4.0 International license.

### Monitoring *C. albicans*-induced inflammasome activation and pyroptosis at single-cell level and in real time.

The uncoupling of hyphal morphogenesis and the ability to cause macrophage death seen in the *mmm1* mutant was striking. Previous studies have reported on mutants that had much milder phenotypes (13), or macrophage death was assessed only at one, early time point (4 or 5 h postinfection) (14, 19, 35). NLRP3 inflammasome-dependent pyroptosis is a dominant mechanism of *C. albicans*-induced macrophage death early in infection (13, 14). The severe and prolonged defect in macrophage killing by the *mmm1* mutant hyphae, coupled with the results from the work of Becker et al. showing that the *mmm1* mutant is avirulent in the murine systemic candidiasis model (24), suggested that the NLRP3 inflammasome response is not only regulated on the basis of fungal morphotype but more sensitively tailored to the pathogenicity of the *C. albicans* strain. We reasoned that the *mmm1* mutant might be a useful tool to understand how this could be achieved.

**FIG 3:**
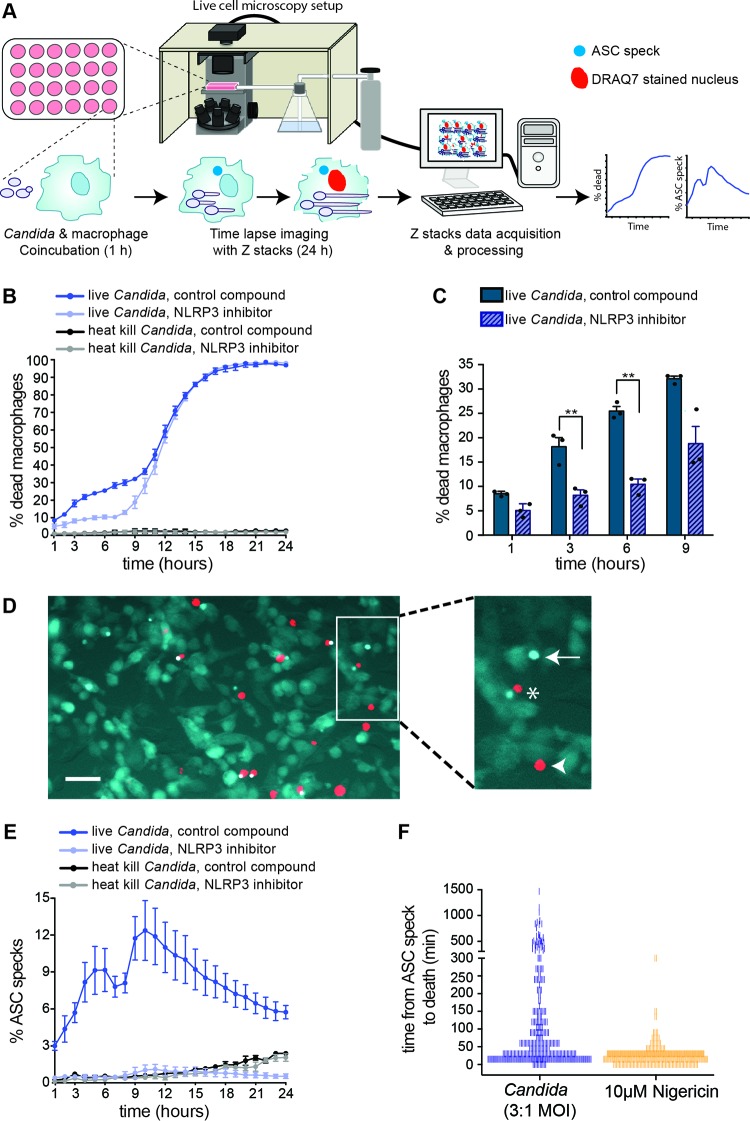
Real-time, single-cell-resolution analysis of *C. albicans* triggering the NLRP3 inflammasome and macrophage pyroptosis. (A) Live-cell imaging assay. ASC-Cerulean macrophages were infected with 3 *Candida* cells per macrophage. The assays were performed in the presence of the NLRP3 inhibitor MCC950 or the inactive small molecule MCC6642 (25), used at 10 µM concentrations. For all panels, the data shown here are the same as the data for the complemented mutant strain (*mmm1+MMM1*) and heat-killed *Candida* in Fig. 4. We show them here separately for clarity of the assay. These experiments were performed in the presence of methionine and cysteine in the medium for direct comparison with the *mmm1* mutant, as shown in Fig. 4. (B) Macrophage cell death quantified from live-cell imaging in the same experiments as ASC speck formation in panel E. Shown are averages from 3 biological repeats and the standard error. See also Movies S3 and S4 in the supplemental material. (C) Bar graph of selected time points from the curves in panel B, with each experimental data point shown in the scatter plot overlay. Statistical significance was calculated by paired *t* test. **, *P* < 0.01. (D) Representative image of ASC speck formation upon recognition of *C. albicans*. Bar, 40 µm. Asterisk, macrophage pyroptosis detected by DRAQ7 as red nuclear fluorescence, in proximity to an ASC-Cerulean speck. Arrow, macrophage displaying inflammasome activation but not yet dead. Arrowhead, pyroptosis-independent death. (E) Quantification of ASC speck formation over time. Shown are averages from 3 biological repeats and the standard error. See also Movies S3 and S4. (F) Time to macrophage death following inflammasome activation by *Candida* or treatment with 10 µM nigericin. Individual macrophages (*n* = 300) were monitored from the moment of ASC speck formation until the appearance of DRAQ7 fluorescence. Each tally represents a single macrophage count and is reported in 15-min increments at the lower end of the scale.

So far, inflammasome activation by fungal pathogens has been studied only in bulk macrophage populations. These lack dynamic spatiotemporal resolution and do not allow for a direct correlation between hyphal morphogenesis, inflammasome activation, and macrophage cell death in response to infection. To understand these processes in greater detail, we established live-cell imaging of *Candida*-induced NLRP3 inflammasome activation and pyroptosis at the single-cell level, in real time, and over the entire interaction time of approximately 24 h (i.e., until essentially all macrophages are killed by *C. albicans*) (Fig. 3A). This allowed us to study the process at unprecedented resolution. For this, we utilized macrophages expressing fluorescently labeled inflammasome subunit ASC (ASC-Cerulean) (36, 37). Under default conditions, ASC is uniformly dispersed in the cytoplasm, but it becomes concentrated in a single speck upon inflammasome activation. The involvement of the NLRP3 inflammasome was addressed by using MCC950, the novel small-molecule inhibitor of NLRP3 (25). Control samples were treated with an inactive compound, MCC6642 (25). Macrophages treated with heat-killed *Candida* and the compounds MCC950 and MCC6642 survived normally in the assay, demonstrating that these molecules do not have adverse effects on macrophage cell survival. ASC speck formation was monitored dynamically over time by live-cell microscopy, and macrophage cell death was monitored simultaneously using the membrane-impermeant DNA-staining dye DRAQ7. Initial experiments revealed that imaging in one plane of focus was not sufficient to capture all ASC speck formation events. Therefore, to ensure that all forming ASC specks were captured in multiple planes of focus, z-stacks spaced 8.5 µm part, totaling 42.5 µm, were taken in time-lapse images.

10.1128/mSphere.00074-16.6 Movie S3  ASC-Cerulean macrophages infected with the control *C. albicans* strain (*mmm1+MMM1*), under control conditions (treatment with the inactive compound MCC6642). The file was created as described for Movie S1. Download Movie S3, AVI file, 2.7 MB. Copyright © 2016 Tucey et al. 2016 Tucey et al. This content is distributed under the terms of the Creative Commons Attribution 4.0 International license.

10.1128/mSphere.00074-16.7 Movie S4  ASC-Cerulean macrophages infected with the control *C. albicans* strain (*mmm1+MMM1*) and treated with the NLRP3 inhibitor MCC950. The file was created as described for Movie S1. Download Movie S4, AVI file, 2.6 MB. Copyright © 2016 Tucey et al. 2016 Tucey et al. This content is distributed under the terms of the Creative Commons Attribution 4.0 International license.

Given that ASC-Cerulean is an immortalized cell line in which NLRP3 and ASC are overexpressed (37), it was important to optimize the infection conditions to closely mimic the response observed in primary BMDMs. Optimization identified that at a multiplicity of 3 *Candida* cells to 1 macrophage, ASC-Cerulean-expressing macrophages behaved similarly to primary BMDMs (13), as 20 to 30% of macrophages were killed in the first 9 h of infection, and all host cells were dead by 24 h (Fig. 3B and C). Infection with *C. albicans* readily caused ASC speck formation (labeled with an arrow), followed by death as determined by the appearance of red, DRAQ7-stained nuclei in the same cell (labeled with an asterisk) (Fig. 3D; see also Movie S3 in the supplemental material). Some macrophages died without ASC speck formation within 9 h postinfection (Fig. 3D, arrowhead), in line with our observation that inactivation of pyroptosis does not block all *C. albicans*-induced macrophage death in the early stage of infection (13). The number of ASC specks increased over time and peaked at 10 h postinfection, after which inflammasome activation stopped (Fig. 3E).

Some ASC specks are lost shortly after macrophage death, meaning that the total number of ASC speck-positive macrophages can be underestimated if considering only those cells that are positive at any given time point. Therefore, to have a clearer estimate of the total number of ASC speck-positive macrophages (i.e., the total number of macrophages that activated the inflammasome), we counted the percentage of dead macrophages that previously displayed an ASC speck during the first 10 h postinfection (*n* = 200). Based on this, we estimated that ≈22% of macrophages contained an ASC speck during the first 10 h of infection; in other words, inflammasome activation by *C. albicans* is heterogeneous and occurs in only a portion of infected macrophages even in this relatively long time course of 10 h. Approximately ≈30 to 35% of macrophages are killed within the first 10 h (Fig. 3B), showing that the majority of DRAQ7-positive macrophages at this time had induced pyroptosis via the NLRP3/ASC/caspase-1 inflammasome. In addition to heterogeneous inflammasome activation, large differences were observed between individual macrophages in the timing of macrophage death post-ASC speck formation, ranging from 15 min to 24 h (Fig. 3F). This means that a small number of macrophages did not readily die after the inflammasome had been activated, but rather that death was observed only after the transition into phase 2 (pyroptosis-independent) death. As a control, we treated macrophages with the bacterial toxin nigericin, a known activator of the NLRP3 inflammasome, and observed immediate and more uniform ASC speck formation in the macrophage population, and in the majority of macrophages death occurred within 45 min (Fig. 3F). The NLRP3 inhibitor MCC950 blocked *C. albicans*-induced inflammasome activation and pyroptosis (Fig. 3E; see also Movie S4 in the supplemental material), and, accordingly, macrophage killing by *C. albicans* in the first 9 h of interaction was significantly reduced (Fig. 3B and C). Consistent with MCC950 blocking *C. albicans*-induced pyroptosis, the macrophage killing curve obtained in the presence of MCC950 closely mimics what we previously observed with *C. albicans* infecting macrophages that are inactivated for the pyroptotic caspases 1 and 11 (13).

### Inflammasome activation in response to the *mmm1* mutant.

**FIG 4:**
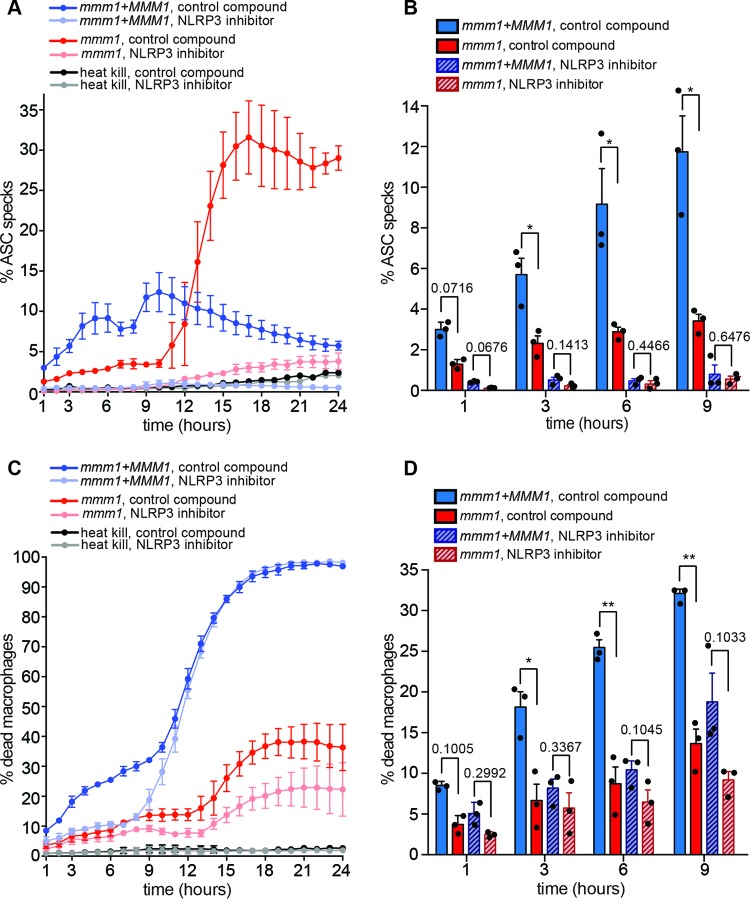
Inflammasome activation in response to the *mmm1* mutant is indicative of a signal threshold response. The movies are uploaded as Movies S3 and S4 (control strain without or with the NLRP3 inhibitor) and Movies S5 and S6 (*mmm1* mutant strain without or with the NLRP3 inhibitor) in the supplemental material. The data with the control and heat-killed *Candida* presented here are the same as those in Fig. 3. In all experiments, the control and the *mmm1* mutant strain were analyzed in parallel for direct comparison. (A) Quantification of ASC speck formation over time, in the presence of control (inactive) compound or the NLRP3 inhibitor MCC950. Macrophages infected with heat-killed *C. albicans* are negative controls. Shown are averages and the standard errors from 3 independent experiments. (B) Bar graph of selected time points from the curves in panel A, with each experimental data point shown in the scatter plot overlay. *, *P* < 0.05 (unpaired *t* test with Welch’s correction). (C) Quantification of macrophage death by DRAQ7 fluorescence. (D) Bar graph of selected time points from the curves in panel C, with each experimental data point shown in the scatter plot overlay. *, *P* < 0.05; **, *P* < 0.01 (unpaired *t* test with Welch’s correction).

To gain insight into how the inflammasome responds to hyphae produced by the avirulent *mmm1* mutant, we used the assay with ASC-Cerulean-expressing macrophages described above (Fig. 4; of note, the data for the complemented *mmm1+MMM1* strain and heat-killed *Candida* shown in Fig. 4 are the same as the data for these control strains shown in Fig. 3). Upon infection of macrophages with the *mmm1* mutant, inflammasome activation was severely delayed for up to 10 h (Fig. 4A and B; see also Movie S5 in the supplemental material). As in BMDMs, this was accompanied by lower rates of cell death of mutant-infected macrophages (Fig. 4C and D). Treatment with the NLRP3 inhibitor abrogated the difference between the *mmm1* mutant and the control strain in macrophage killing in the first 9  h (Fig. 4C and D; see also Movie S6). This shows that reduced macrophage killing is largely due to the inability of the mutant to trigger NLRP3-dependent pyroptosis for a prolonged time.

10.1128/mSphere.00074-16.8 Movie S5  ASC-Cerulean macrophages infected with the *mmm1* mutant strain under control conditions (treatment with the inactive compound MCC6642). The file was created as described for Movie S1. Download Movie S5, AVI file, 1.7 MB. Copyright © 2016 Tucey et al. 2016 Tucey et al. This content is distributed under the terms of the Creative Commons Attribution 4.0 International license.

10.1128/mSphere.00074-16.9 Movie S6  ASC-Cerulean macrophages infected with the *mmm1* mutant and treated with the NLRP3 inhibitor MCC950. The file was created as described for Movie S1. Download Movie S6, AVI file, 1.9 MB. Copyright © 2016 Tucey et al. 2016 Tucey et al. This content is distributed under the terms of the Creative Commons Attribution 4.0 International license.

Unexpectedly, after 10 h of infection, macrophages infected with the *mmm1* mutant showed a sharp increase in ASC speck formation, surpassing what is seen with the control strain (Fig. 4A). More than 30% of macrophages contained ASC specks by 16 h (the highest ASC speck value observed was 31.58%). This exacerbated ASC speck formation was almost entirely blocked by the NLRP3 inhibitor MCC950 (Fig. 4A), demonstrating that it is due to NLRP3 activation. The increase in ASC specks was mirrored by an increase in macrophage cell death starting at 13 h and reaching ≈35% by 24 h (the highest observed death value was 38.29% [Fig. 4C]). The NLRP3 inhibitor partially rescued *mmm1*-induced macrophage death (Fig. 4C and D).

**FIG 5:**
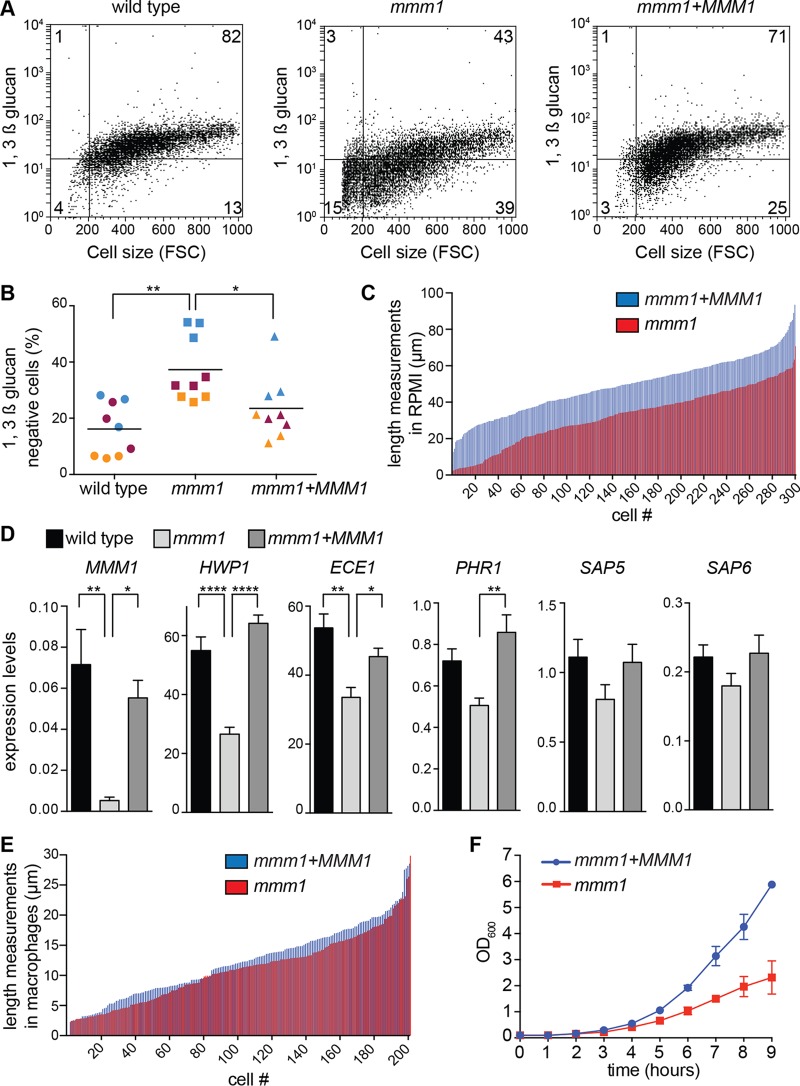
ERMES impacts on the exposure of pathogen-associated molecular patterns during hyphal growth. (A) Hyphae were grown for 3 h in RPMI medium under repressive conditions, and surface-exposed 1,3-β-glucan was analyzed by flow cytometry following staining with the 1,3-β-glucan antibody. The experiment was repeated 3 times with equivalent results (see also panel B). Shown are representative dot plots from one biological replicate for each strain, plotting 1,3-β-glucan staining versus cell size (forward scatter [FSC]), with the percentage of cells shown for each quadrant. The upper left and right quadrants are glucan-positive cells, while the lower left and right quadrants are glucan-negative cells. (B) Percentage of cells that are negative for exposed 1,3-β-glucan. *n* is 9 from 3 independent experiments with 3 biological replicates each. The biological replicates analyzed together are shown by the same color. The line represents the mean. *, *P* < 0.05; **, *P* < 0.01 (one-way analysis of variance, followed by Tukey’s multiple-comparison test). (C) Hyphal length after 3 h in RPMI medium. Each measured cell is depicted by a line, with length shown on the *y* axis and the measurements ranked in order from smallest to largest on the *x* axis. Three biological replicates were performed. One repeat is shown here, and the other two are in Fig. S7B in the supplemental material. (D) Hyphal gene expression after 3 h in repressive RPMI medium. Shown is the ratio of gene expression normalized to *SCR1*. Error bars represent standard errors of the averages from 6 biological replicates assayed in 2 independent experiments. *, *P* < 0.05; **, *P* < 0.01; ****, *P* < 0.0001 (one-way analysis of variance followed by Tukey’s multiple-comparison test). (E) Hyphal length distribution in macrophages following 1 h of coincubation and washes. Data were analyzed in 2 independent experiments with equivalent results (*n* = 200 fungal cells per strain). One experiment is shown here, and the other is shown in Fig. S7C in the supplemental material. (F) Growth at 37°C in repressive medium. Shown are the averages and the standard errors of the means from 3 biological replicates assayed in the same experiment. OD_600_, optical density at 600 nm.

The kinetics of ASC speck formation in infections with the *mmm1* mutant shows that, in response to a strain of reduced pathogenicity, inflammasome activation can be delayed by several hours. Importantly, inflammasome activation in response to the *mmm1* mutant was not blocked and eventually occurred sharply. The kinetics suggested that signals derived from hyphae activate the inflammasome by a threshold mechanism, reminiscent of the recently described “digital” mode of activation for caspase-1 in response to various signals (38). In this scenario, in infections with the *mmm1* mutant, the threshold is reached much later than in controls. Mitochondrial activity is needed for cell wall integrity in *C. albicans* (39–41; reviewed in references 5 and 6), and cell wall components, including 1,3-β-glucan and cell wall mannosylation, correlate with inflammasome activation and pyroptosis (13, 19, 42–44). We therefore hypothesized that the *mmm1* mutant shows delayed cell wall restructuring during hyphal morphogenesis, leading to reduced numbers of exposed cell surface molecules that could be providing the signal for inflammasome activation. Consistent with this proposition, the percentage of cells that were negative for exposed 1,3-β-glucan was significantly higher in hyphal cultures of the *mmm1* mutant than in controls (Fig. 5A and B). We further noticed that the population of shorter 1,3-β-glucan-negative cells was larger in the *mmm1* mutant (Fig. 5A, lower left and right quadrants of dot plots). Since the formation of hyphae is linked to NLRP3 inflammasome activation, presumably due to changes to the fungal cell surface or cell physiology that provide the required signal, we addressed the expression of some of the genes induced in hyphal cells compared to yeast. Figure 5D shows that the expression levels of hypha-specific genes *HWP1* and *ECE1* and the cell wall glycosidase *PHR1* were reduced in a statistically significant manner in *mmm1* mutant hyphae (Fig. 5D). The *mmm1* mutant hyphae were of normal morphology (Fig. 2E), but our data in Fig. 5A suggested that the mutant displayed reduced hyphal elongation. Measurements showed that the distribution of hyphal lengths was similar to that in controls, but there was a shift toward shorter lengths in macrophages and a very clear difference in the length of hyphal filaments in *in vitro* cultures (Fig. 5C and E; see also Fig. S7B and C in the supplemental material). The more pronounced defect in hyphal lengths *in vitro* than in macrophages is due to a longer time of hyphal growth *in vitro* (in macrophages, cell lengths were determined after 1 h of coincubation, as it is difficult to determine cell lengths at later time points when extensive hyphal growth occurs; *in vitro*, the length of the filaments was determined at 3 h post-induction of hyphal growth). At host temperature (37°C), growth retardation for the *mmm1* mutant was observed after 4 h (Fig. 5F). Collectively, these results show that *MMM1* is not required for hyphal morphogenesis *per se*. However, *MMM1* is needed to establish wild-type levels of hyphal growth, elongation, and cell wall remodeling that are required to rapidly reach the signal threshold for inflammasome activation by *C. albicans* and enable fungal escape through pyroptosis.

## DISCUSSION

### Roles of ERMES and mitochondrial morphology in fungal virulence.

Here, we report the first detailed characterization of the ERMES complex in a pathogenic fungal species, showing how this mitochondrial hub is important for the ability of *C. albicans* to kill macrophages for immune escape. Following conditional inactivation of ERMES, changes to mitochondrial morphology were seen first. Moreover, they could be uncoupled from fitness, respiratory, and lipid perturbations that were seen only after longer-term inactivation of the complex. Therefore, our data suggest that the primary role of *C. albicans* ERMES is in maintaining mitochondrial shape. Our characterization suggests that wild-type mitochondrial network shape is important for differentiating hyphae that can trigger pyroptotic macrophage death. While the *mmm1* mutant was able to form hyphae both in liquid tissue culture medium and in macrophages, these mutant hyphae were unable to trigger NLRP3-dependent pyroptosis for a very long time of approximately 10 h. Hyphal filaments made by the *mmm1* mutant were of normal morphology, and overall length distribution was similar to that of the control, but with a shift toward shorter lengths, indicating reduced hyphal growth and elongation. Compared to the control, fitness differences were not obvious for several hours in macrophages. In wild-type filaments, mitochondrial tubules extend throughout the hyphal cell, while in the ERMES mutants, the “giant” spherical mitochondrial morphology means that large parts of the hyphal filaments are devoid of mitochondria (see Fig. S7A in the supplemental material). Proper mitochondrial distribution in the filaments could serve to power hyphal elongation, as hyphal growth depends more prominently on mitochondrial metabolism than does yeast growth (45). The mitochondrial defects in the *mmm1* mutant further had more important contributions to growth at 37°C than at 30°C with glucose as the carbon source (Fig. 1 and 5), and the *mmm1* mutant was unable to grow on glycerol plates at 37°C, consistent with mitochondrial dysfunction (data not shown). Hyphal populations from the *mmm1* mutant displayed overall surface changes, with smaller amounts of exposed 1,3-β-glucan and moderately reduced expression of *HWP1* and *PHR1* encoding a hypha-specific cell wall adhesin and a cell wall-remodeling enzyme, respectively, as well as *ECE1*, a hypha-specific gene that was recently shown to encode a toxin that can cause damage to epithelia (46). These changes in cell wall structure and hyphal gene expression are likely to lead to severely delayed inflammasome activation, delayed pyroptosis, and lack of fungal escape, as both glucan and cell wall protein mannosylation have been implicated in inflammasome activation and pyroptosis by *C. albicans* (13, 19, 42–44). Importantly, while severely delayed, inflammasome activation by the *mmm1* mutant eventually occurred and it paradoxically surpassed what is seen in macrophages infected with the control strain. Exacerbated inflammasome activation was dependent on NLRP3, and all host killing by the *mmm1* mutant at later time points in infection was by pyroptosis. To our knowledge, the *mmm1* mutant phenotype of delayed inflammasome activation and pyroptosis followed by an exacerbated response has not been reported for any other *C. albicans* mutants that showed inflammasome activation defects. This illustrates the power of our live-cell microscopy assay to dynamically monitor inflammasome activation and pyroptosis in parallel. The implications of this result for the mechanism of inflammasome activation by *C. albicans* are further discussed below. In addition to being unable to rapidly activate macrophage pyroptosis, the *mmm1* mutant was also unable to trigger the pyroptosis-independent phase 2 of macrophage killing, as almost all macrophage death in mutant infections was by pyroptosis, after which death plateaued. While the *mmm1* mutant survived intracellularly in macrophages for extended periods of time and formed hyphae, growth was reduced later in infection. Therefore, the presence of even substantial intracellular hyphae is not sufficient to trigger the phase 2, caspase-1-independent form of cell death; this requires rapidly growing, persistent filaments. Collectively, our data show that ERMES coordinates short-term survival strategies of *C. albicans* by triggering rapid macrophage pyroptosis, with longer-term effects by ensuring optimal growth at host temperature and the ability to trigger multiple mechanisms of macrophage cell death. Our characterization further explains the role of *MMM1* in systemic virulence in mutant library screens (24).

### Insight into the NLRP3 inflammasome response to *C. albicans.*

Characterization of the ERMES mutant, coupled with real-time single-cell imaging of inflammasome activation, showed that (i) NLRP3 inflammasome activation by *C. albicans* is heterogeneous in the macrophage population; (ii) the inflammasome response is sensitively tailored to hyphal growth, and we propose that this is achieved via threshold activation; (iii) macrophages can be sensitized to NLRP3 inflammasome activation; and (iv) the new NLRP3 inhibitor MCC950 blocks inflammasome activation and pyroptosis following *Candida* infection. MCC950 is promising for treating diseases associated with pathogenic inflammation (25), and based on our data, we suggest that MCC950 could be explored for modulating *Candida*-induced inflammation that might be contributing to disease.

Although the majority of macrophages were infected at the multiplicity of infection (MOI) used (3 *Candida* cells per macrophage), only a proportion of up to 22% of them activated the NLRP3 inflammasome. We noticed that the number of *C. albicans* cells phagocytosed by a single macrophage varied in the population. This could be related to distinct inflammasome activation in individual macrophages, and future experiments will address this. Our observation is in line with a recent report showing that another NLRP3-inflammasome activator, silica crystals, caused caspase-1 activation in only a fraction of host cells (38). In contrast, treatment with the potassium ionophore nigericin triggers a more uniform NLRP3 inflammasome activation (37). The molecular mechanism leading to NLRP3 inflammasome activation by any stimuli remains ill defined, but it is thought that lysosome rupture contributes in the case of silica crystals and *C. albicans* hyphae. Activation by nigericin could be more direct due to rapid potassium efflux. Therefore, signals derived from lysosomes might require a threshold to activate the NLRP3 inflammasome that is reached distinctly in individual macrophages and could depend on signals derived from the pathogen and/or on factors present in only a subpopulation of macrophages. Activation of the NLRP3 inflammasome by *C. albicans* is a double-edged sword, as it triggers lytic pyroptosis that enables escape but also activates antifungal responses. Heterogeneous responses in infected macrophages could modulate these contrasting processes, with potential benefits to pathogen or host.

**FIG 6:**
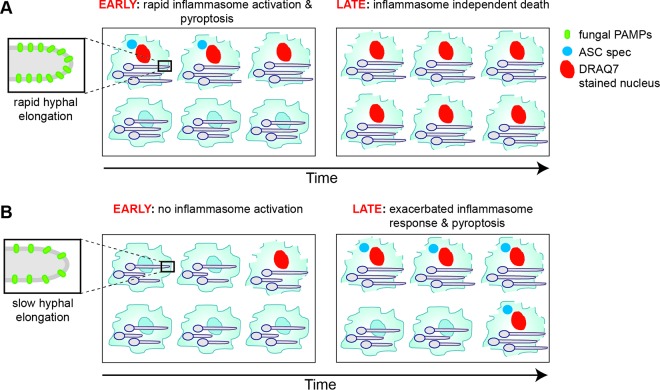
Model of inflammasome activation by *C. albicans* strains of distinct hyphal growth. In strains with robust filamentation and high virulence, inflammasome activation occurs rapidly upon phagocytosis by macrophages, leading to host cell pyroptosis. In infection by a strain with reduced virulence (*mmm1* mutant), a longer time is needed to trigger the inflammasome, but eventual sensitization of host cells occurs, and inflammasome activation is seen in a larger number of macrophages in the population than that with the control strain with more robust filamentation. These differences could be explained by a signal threshold mode of inflammasome activation that depends on hyphal growth, cell wall remodeling, and exposure of fungal pathogen-associated molecular patterns (PAMPs).

The dynamics of inflammasome activation by the *mmm1* mutant showed that infection with a less virulent strain leads to a long delay in inflammasome activation despite the formation of hyphae. Importantly, the *mmm1* mutant hyphae eventually triggered the inflammasome response (Fig. 4 and model in Fig. 6). The kinetics of the inflammasome response to the *mmm1* mutant is consistent with a signal threshold response. Another possible explanation is that, at later time points postphagocytosis, the *mmm1* mutant hyphae express alternative signals that activate the NLRP3 inflammasome by a different mechanism than what is observed with the control strain. We favor the signal threshold model, as the *mmm1* mutant hyphae displayed quantitative changes in hyphal growth/filament length, in surface-exposed 1,3-β-glucan, and in the expression of hyphal genes. Consistent with our proposition for a signal threshold model are recent data of caspase-1 activation in response to *Salmonella*, or signals such as the NLRP3 activator silica, which showed “digital” or threshold signaling (38). Host responses to *C. albicans* in epithelial cells have been shown to depend on fungal cell numbers, also suggesting threshold signaling (47). Our data suggest that, to reach the threshold for NLRP3 inflammasome activation, hyphae need to elongate persistently, accompanied by cell wall remodeling and exposure of pathogen-associated molecular patterns (PAMPs), such as 1,3-β-glucan or mannosylated cell wall proteins (Fig. 6). Relevant to this is a recent report of a distinct structure of glucan derived from *C. albicans* hyphae compared to yeast and more potent stimulation by hyphal glucan of interleukin-1β (IL-1β) secretion as a proxy for caspase-1 activation (48). As discussed in a recent review, threshold mechanisms of signaling allow for sensitive responses tailored to the level of threat, thereby minimizing noisy inflammatory responses that could be detrimental to the host (49). In the case of *C. albicans*, this means that not only does the inflammasome discriminate yeast from hyphal morphology (16) but the response is more sensitively fine-tuned to hyphal growth levels, which could allow for tight regulation.

The kinetics of inflammasome activation by the *mmm1* mutant showed sensitization of the response and exacerbated activation after a prolonged delay (Fig. 4). This result should be considered in the light of differences in filamentation robustness, growth rates, and virulence potential within a collection of *C. albicans* clinical isolates (50). In this set, SC5314, the parent of most laboratory strains, including our own, is on the extreme end, being a highly filamentous strain (50). It could be that the dynamics of inflammasome activation that we see with the *mmm1* mutant, with the prolonged delay, sensitization, and hyperactivation, is representative of some clinical strains of *C. albicans* as well as of the response at a low multiplicity of infection, as is likely to be the case in clinical situations.

### Conclusions and outlook.

Our study lays the foundation for exploring the roles of ERMES, and mitochondrial dynamics processes more generally, in fungal immune evasion and virulence across diverse human fungal pathogens. Evidence suggests that disrupting mitochondrial morphology might be a global antifungal strategy. In addition to *C. albicans* (this study and reference 51), mitochondrial morphology has been implicated in virulence-related processes in *Cryptococcus gattii* (52) and *Aspergillus fumigatus* (53). Due to its absence in humans (23) and a key role in maintaining the tubular mitochondrial network structure, ERMES is particularly promising for antifungal drug discovery. Our data further develop a new understanding of how the dynamic inflammasome response to *C. albicans* is fine-tuned to reflect the pathogenic state of the fungus. The single-cell-resolution, parallel live-cell imaging of the inflammasome response and pyroptotic death that we established can be extended in the future to multiple fungal strains, as well as studying the impact of genetic and pharmacological manipulation of host and pathogen pathways on immune interactions.

## MATERIALS AND METHODS

### *C. albicans* strains and growth conditions.

The *C. albicans* strains used in this study are derivatives of BWP17 and are listed in Table S1 in the supplemental material. Primers for strain construction are listed in Table S2. Methods for strain construction and growth conditions are detailed in Text S1 in the supplemental material.

10.1128/mSphere.00074-16.1 Text S1  Supplemental materials and methods. Download Text S1, PDF file, 0.1 MB. Copyright © 2016 Tucey et al. 2016 Tucey et al. This content is distributed under the terms of the Creative Commons Attribution 4.0 International license.

### Microscopy.

Detailed microscopy methods are given in Text S1 in the supplemental material. Images were taken with a 100× objective using an Olympus BX60 fluorescence microscope equipped with Spot Advanced Software (Spot Imaging, Sterling Heights, MI). Mitochondrial network morphology was imaged following staining with MitoTracker dyes. Hyphal formation was assessed in liquid RPMI medium under repressive conditions (with 2.5 mM methionine and 0.5 mM cysteine), after 3 h at 37°C. The lengths of filaments were measured using Fiji (http://www.fiji.sc/Fiji).

### Macrophage interaction assays.

Experiments involving animals were approved by the Monash University Animal Ethics Committee, in accordance with the guidelines and policies in the Australian code for the care and use of animals for scientific purposes provided by the Australian National Health and Medical Research Council (approval numbers SOBS-2010-M-49 and MARP-2011-086). Murine bone marrow-derived macrophages (BMDMs) were obtained essentially as described in reference 13; please see Text S1 in the supplemental material for a detailed description. For these experiments, ERMES gene repression was initiated by patching *C. albicans* colonies overnight on repressive medium plates at 30°C, after which *C. albicans* cells were resuspended in phosphate-buffered saline (PBS) and counted and macrophages were infected at a multiplicity of infection (MOI) of 6:1 (*Candida* cells to macrophage). Four biological replicates were performed, and the data were analyzed in GraphPad Prism. The immortalized mCerulean-tagged ASC inflammasome reporter macrophages were a gift from Eicke Latz (36). Live-cell imaging was set up as described above, and the MOI was 3:1 (*Candida* cells to macrophage). The acquisition of time-lapse images, methods for processing, and counting of ASC speck formation are described in Text S1 in the supplemental material. All macrophage experiments (with BMDMs and ASC-Cerulean macrophages) were done in the presence of 2.5 mM methionine and 0.5 mM cysteine in the medium to allow for *MMM1* gene repression. Heat-killed *Candida* cells were incubated at 80°C for 1 h prior to addition to macrophages. For experiments including drug treatments, 10 µM MCC950 or 10 µM MCC6642 (both made as 10 mM stocks in dimethyl sulfoxide [DMSO]) or 10 µM nigericin (Invitrogen) was included at the same time as addition of *Candida*.

### Mitochondrial isolation and protein import assays.

Isolation of mitochondria and mitochondrial protein import assays were performed as previously described (54). In order to improve detection, ImageJ was used to apply a contrast alteration to the entire phosphorimage scan, in a manner that maintains the linear relationship of the gray tones in the image.

### Quantitative RT-PCR analysis.

All primers used for quantitative RT-PCR are listed in Table S3 in the supplemental material, and some are further described in references 55 and 56. Growth conditions and experimental setup are described in the figure legends, and the methods are further detailed in the supplemental material. Data analysis was done using LinReg software (57).

### Phospholipid analysis.

For lipid extractions, conditional ERMES mutants or homozygous deletion mutants were grown as described in Text S1 in the supplemental material and the figure legends. The procedure for lipid extraction is detailed in Text S1. Lipids were normalized according to protein levels and separated by thin-layer chromatography (TLC). Standards were from Avanti Polar Lipids. Lipids were quantified using the Toolbox module of ImageQuant 1D version 7.0, and background signals were subtracted using the local median method performed by the software.

### Analysis of 1,3-β-glucan by flow cytometry.

1,3-β-Glucan on hyphal cells was quantified as previously described (13).

### Statistical analysis.

Statistical analysis was performed using GraphPad Prism software, and the relevant tests used are indicated in the figure legends. Biological repeats were from cultures obtained from independent colonies of the indicated *C. albicans* strains. For the experiments with bone marrow-derived macrophages, different mice were used for the independent experiments.

10.1128/mSphere.00074-16.3 Tables S1 to S3 (Table S1) *C. albicans* strains used in the study.
(Table S2) Strain construction primers.
(Table S3) Quantitative RT-PCR primers. Download Tables S1 to S3, PDF file, 0.1 MB. Copyright © 2016 Tucey et al. 2016 Tucey et al. This content is distributed under the terms of the Creative Commons Attribution 4.0 International license.
