# Supplementary figures and images for: The Endoplasmic Reticulum-Mitochondrion Tether ERMES Orchestrates Fungal Immune Evasion, Illuminating Inflammasome Responses to Hyphal Signals
Source: mSphere. 2016 May 25;1(3):e00074-16. doi: 10.1128/mSphere.00074-16 (PMC4888881; doi:10.1128/mSphere.00074-16)

Figure S1

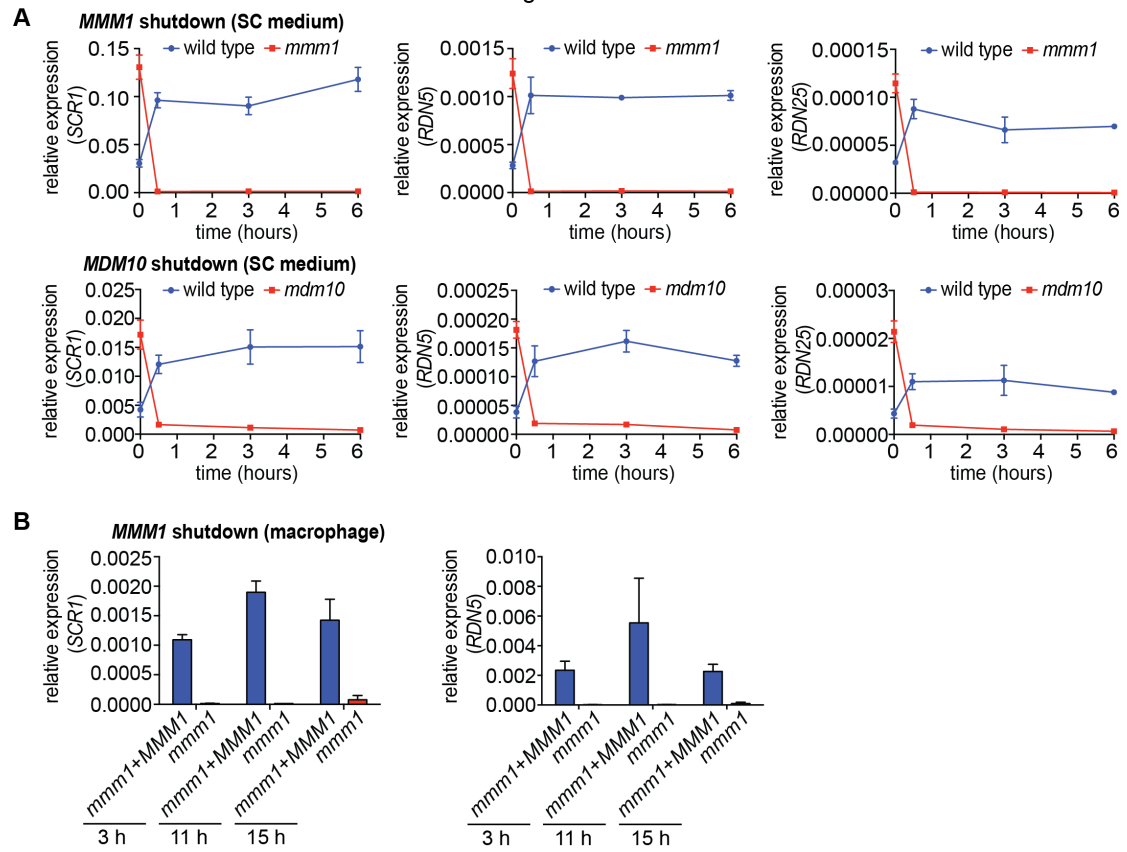

Figure S2

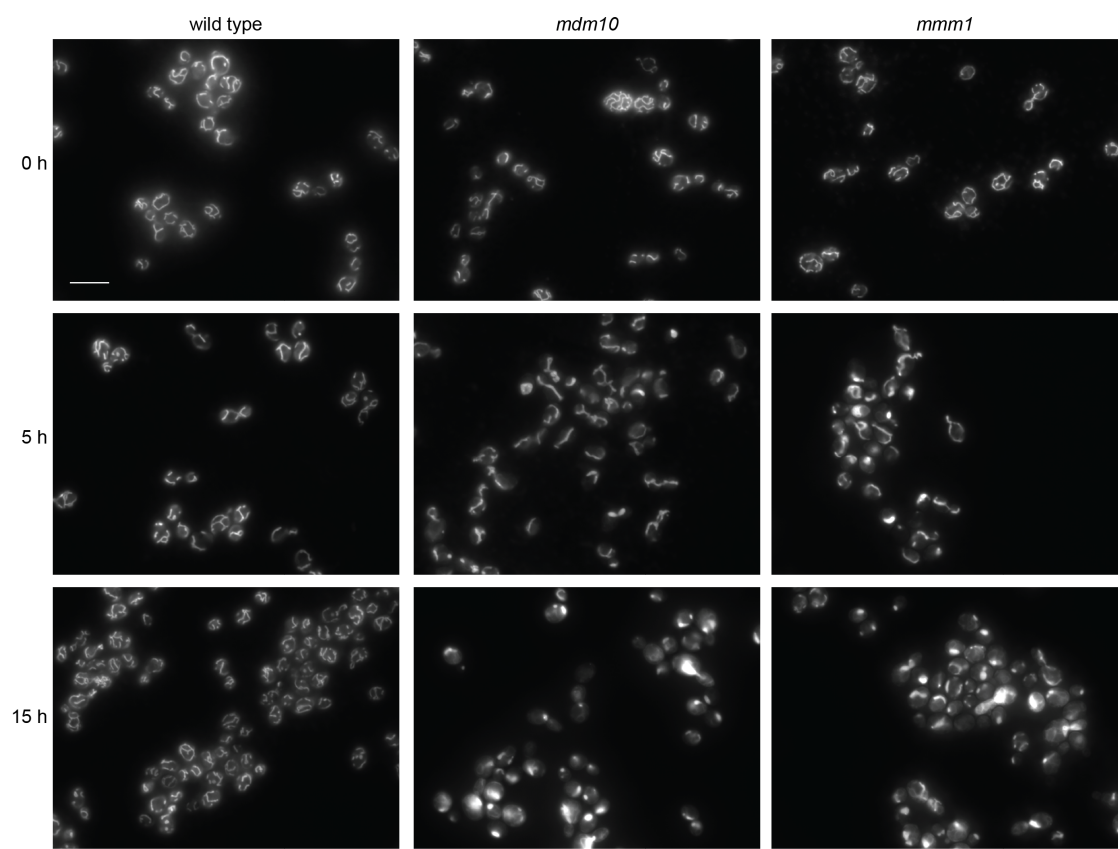

Figure S3

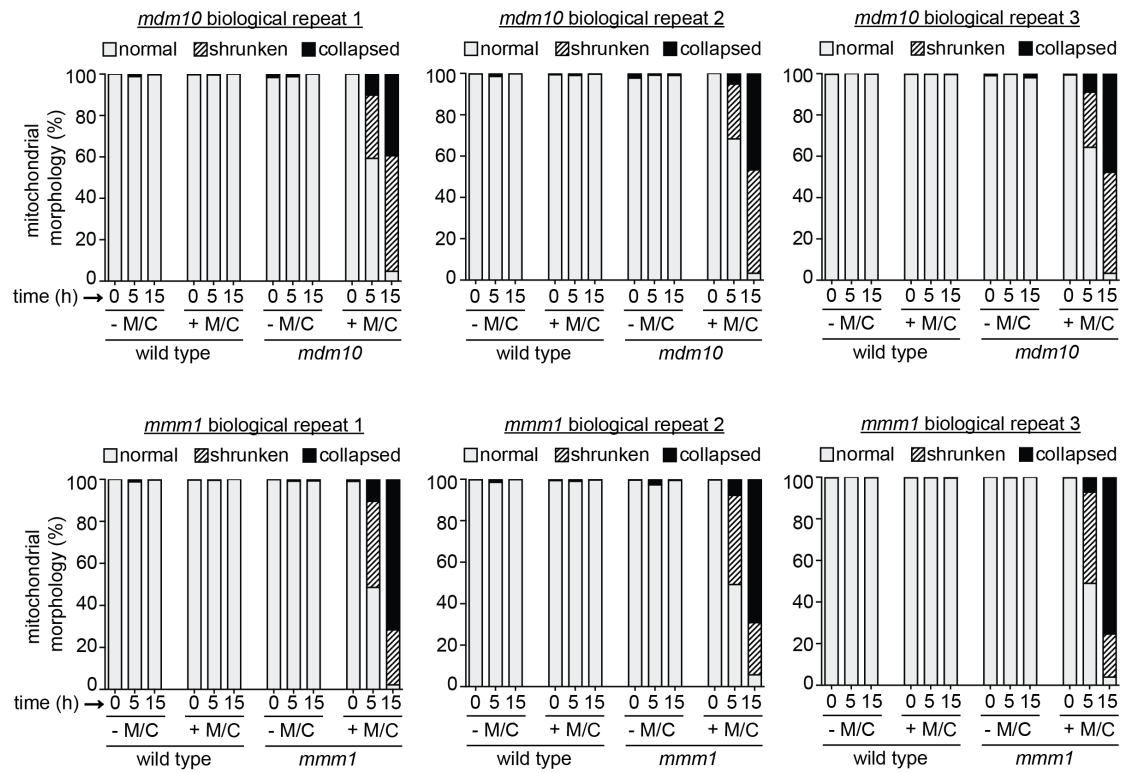

Figure S4

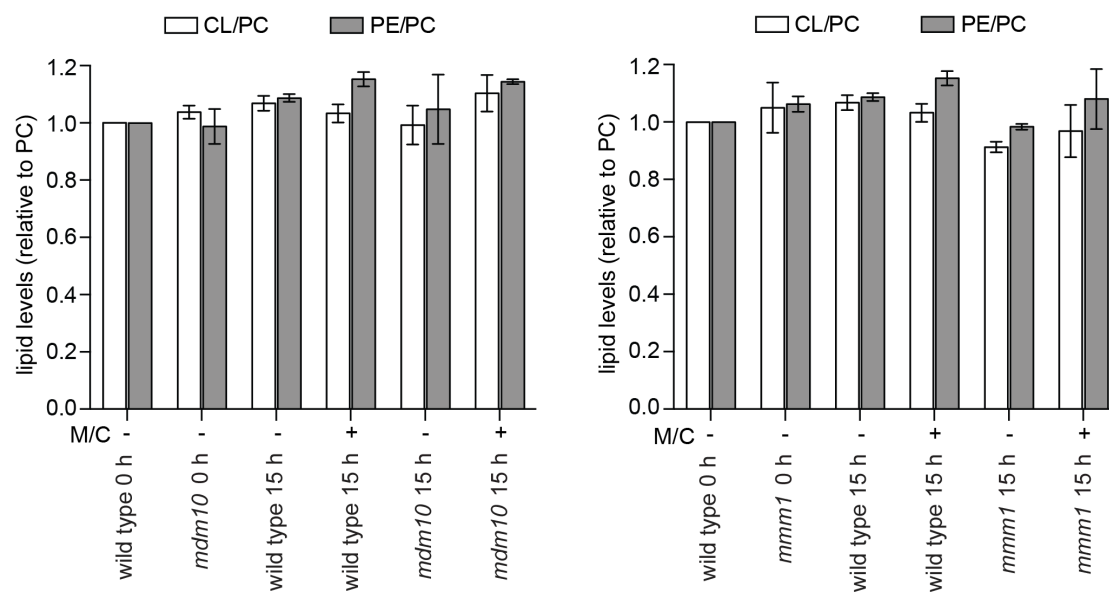

Figure S5

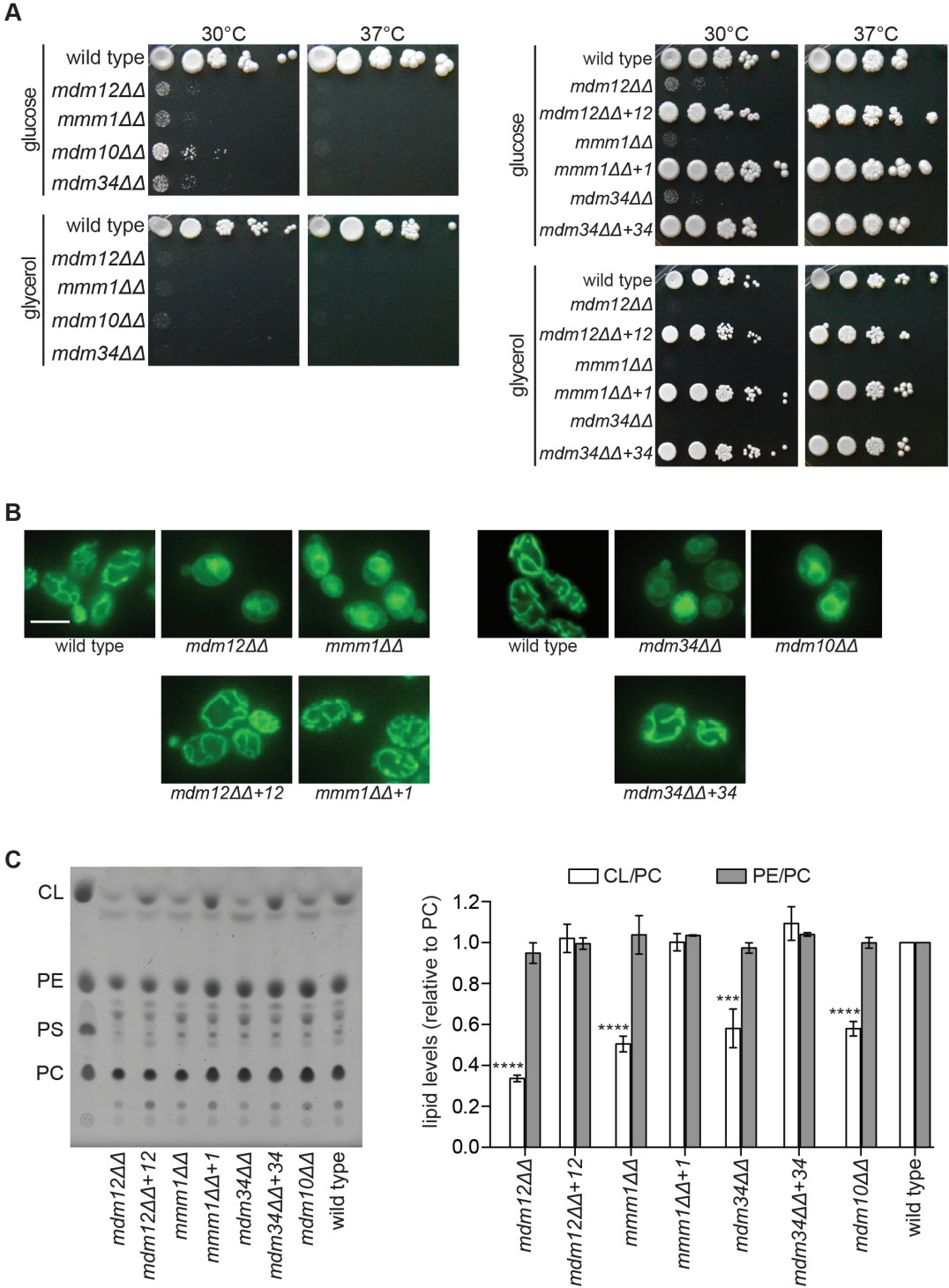

Figure S6

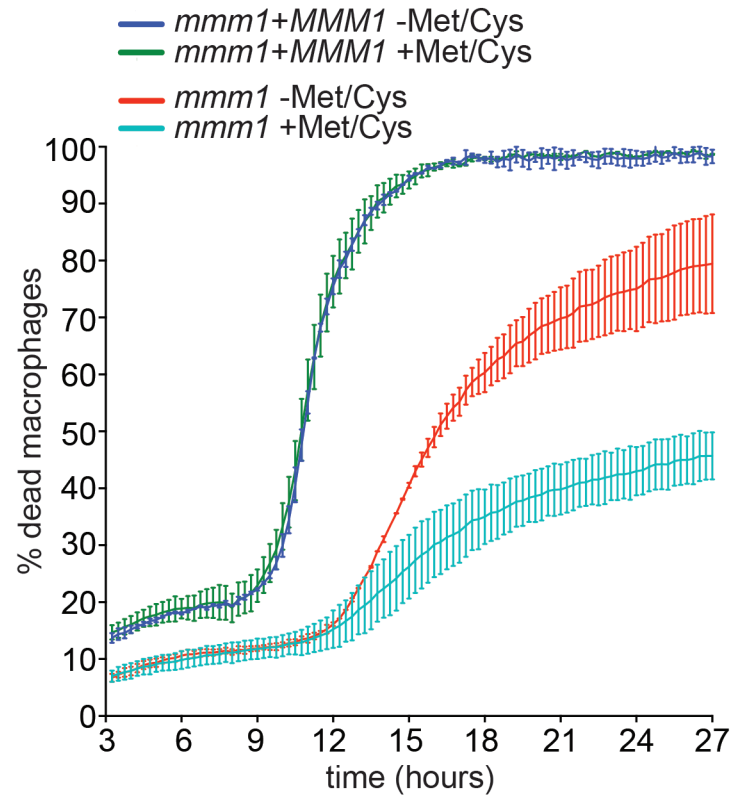

Figure S7

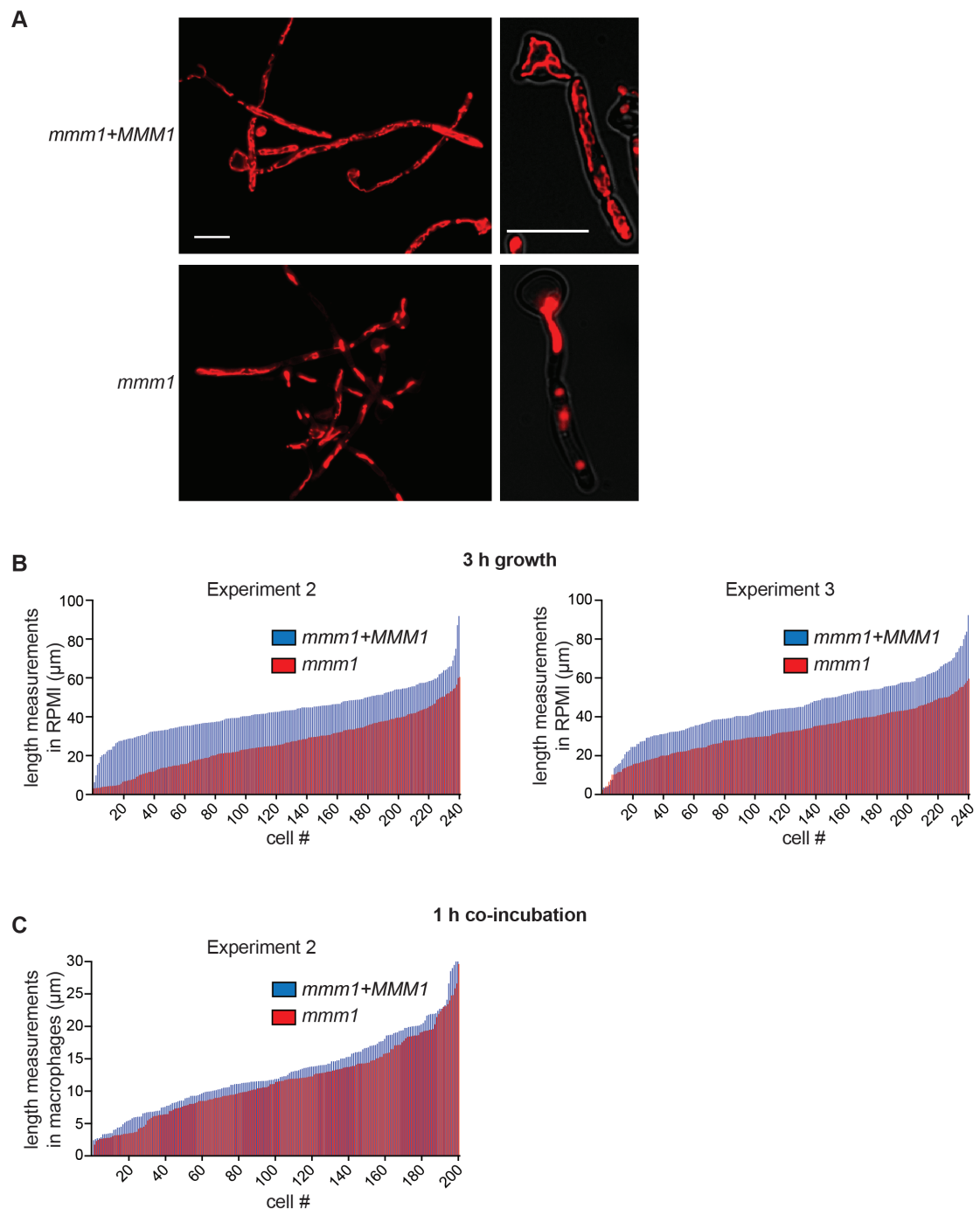

Supplement: Figures S1 to S7 [file sph003162089sf2.pdf]
