# Supplementary material for: The Endoplasmic Reticulum-Mitochondrion Tether ERMES Orchestrates Fungal Immune Evasion, Illuminating Inflammasome Responses to Hyphal Signals
Source: mSphere. 2016 May 25;1(3):e00074-16. doi: 10.1128/mSphere.00074-16 (PMC4888881; doi:10.1128/mSphere.00074-16)
Supplement: Text S1 [file sph003162089s1.pdf]

**Supplementary Methods**

***C. albicans* strains and growth conditions**

The conditional ERMES mutants were constructed by deleting one copy of the gene and placing the other under the control of the *MET3* promoter using standard methods based on PCR and homologous recombination (the *URA3* and *ARG4* markers were used for these constructions). The mutant strains were reverted to histidine prototrophy by integrating the *HIS1*-containing integrative pDDB78 vector. The complemented strain for the conditional *mmm1* mutant was constructed by introducing a wild type *MMM1* gene under its own promoter and terminator into the *HIS1* locus of the mutant, using the pDDB78 plasmid. The ERMES homozygous deletion strains (Fig. S5) were constructed by deleting both gene copies with *ARG4* and *URA3* markers, and the complemented strains constructed as described above. The wild type control strains DAY185 (*URA3*+ *ARG4*+ *HIS1*+) or DAY286 (*URA3*+ *ARG4*+ *his1-)* were used as appropriate.

Strains were grown at 30°C, 200-250 rpm unless otherwise indicated. Rich media was YPD (1% yeast extract, 2% peptone, 2% glucose) supplemented with 80 μg/mL uridine. Synthetic media was made with 0.67 % w/v yeast nitrogen base without amino acids or carbohydrate and with ammonium sulfate (US Biological Y2025) with 2 % glucose (w/v) or 3 % glycerol (w/v) as indicated, and supplemented with 80 μg/mL uridine and the appropriate amino acids. For repressive conditions 2.5 mM methionine and 0.5 mM cysteine were added to the media. For growth curves in Fig. 1, cultures were grown in permissive conditions overnight at 30 °C. At time point 0, cultures were diluted to $OD_{600}$=0.1 in repressive (+ methionine and cysteine) media and growth monitored at $OD_{600}$ at 30 °C. For growth curves in Fig. 5, strains were

grown overnight in repressive conditions at 30 °C. Following dilution to $OD_{600}$=0.1 at time point 0, growth at 37 °C was monitored by measuring $OD_{600}$ over time in repressive (+ methionine and cysteine) media.

***Microscopy***

Fluorescence images of mitochondria were produced using 1 μM MitoTracker Red CMXRos (Life Technologies, M7512) or 1 μM MitoTracker Green FM (Life Technologies, M7514) stock diluted into the growth media to give a final concentration of 0.1 μM and used as per the manufacturer's instructions. Images were taken at 100x objective, 72 DPI resolution (1600 x 1200 pixels) using an Olympus BX60 fluorescence microscope equipped with Spot Advanced Software (http://www.spotimaging.com/software/), and images were subsequently re-sampled for inclusion into 300 DPI resolution figures. For a given experiment containing wild type and mutant cells, all bright field and fluorescence images were taken with the same exposure time and under equivalent brightness and contrast settings. Red or green filters were applied to representative images using the ImageJ software to reflect the dyes used.

For liquid filamentation experiments, strains were grown in repressive conditions at 30 °C overnight, then diluted to $OD_{600}$=0.1 in RPMI media containing 2.5 mM methionine and 0.5 mM cysteine. Hyphal formation was assessed after 3 h of growth at 37 °C at 200 rpm. Hyphal lengths were quantified using Fiji (http://www.fiji.sc/Fiji) following image collection. To measure hyphal lengths, Fiji's freehand tool was used to draw a distance from cell edge to hyphal tip, and this distance measured using Fiji's measure tool, resulting in an output of length in pixels.

Pixel length was converted to µm based on microscope scale bar settings. Hyphae and cells that extended past the edges of images were not quantified.

***Macrophage interaction assays***

For the macrophage interaction assays, single colonies of the *C. albicans* strains were patched on plates repressive for ERMES gene expression (complete synthetic media with uridine, and with addition of 2.5 mM methionine and 0.5 mM cysteine). Strains were grown over night for 12 h at 30 °C. Cells were taken from the plates and resuspended in PBS, followed by counting before addition to macrophages. All macrophage live-cell microscopy experiments were performed in media with addition of 2.5 mM methionine and 0.5 mM cysteine to repress the *MET3p-MMM1* gene in the mutant. The complemented control strain contains the *MMM1* gene under its own promoter integrated in the *HIS1* locus, and is therefore not repressible by methionine and cysteine.

Murine bone-marrow derived macrophages (BMDMs) were obtained by culturing bone marrow cells from 6-8 week old C57BL/6 mice in RPMI 1640 medium supplemented with 12.5 mM HEPES, 15% fetal bovine serum (Serana), 20% L-cell conditioned medium (containing macrophage colony-stimulating factor), and 100 U/mL of penicillin-streptomycin (Sigma) for 7 days, at 37°C + 5% $CO_2$. Macrophages were seeded at a density of 5 x $10^5$ cells/well in 24-well tissue culture plates. Macrophages were stained with 1 µM Cell Tracker Green (Invitrogen) for 20 min in serum-free RPMI 1640. Macrophages were infected with *C. albicans* at a MOI of 6:1 (*Candida*:macrophage). After 1 h of co-incubation, non-phagocytosed fungal cells were removed by washing three times with phosphate-buffered saline (PBS), followed

by staining with 0.6 µM DRAQ7 (Abcam). Live cell imaging was performed on a Leica AF6000 LX epi-fluorescence microscope equipped with an incubator chamber set at 37°C + 5% $CO_2$ and an inverted, fully motorized stage driven by Leica Advanced Suite Application software. Time-lapse images were acquired with bright field, GFP, and Y5 filters every 15 minutes up to 24 h using a 10x/0.8-A objective. To determine the percentages of dead cells, images were analyzed in ImageJ and in MetaMorph (Molecular Devices) using a custom-made journal suite incorporating the count nuclei function to segment and count the number of Cell Tracker Green and DRAQ7 positive cells (adapted from (1)) The data for percentage DRAQ7 positive cells were analyzed in GraphPad Prism.

The immortalised mCerulean-tagged ASC inflammasome reporter macrophages were cultured in RPMI with 12.5 mM HEPES and 10% FBS and seeded at a density of 2.5 x $10^5$ cells/well in 24-well tissue culture plates. Macrophages were infected with *C. albicans* at a MOI of 3:1 (*Candida*:macrophage), and live cell imaging was set up as described above, but with the following adjustments: time-lapse images were acquired with bright-field, CFP, and Y5 filters every 1 hour up to 24h, and z-stacks spaced 8.5 µm totaling 42.5 µm were taken to account for all ASC specks across macrophages in multiple planes of focus. These image slices were subsequently deconvolved using ImageJ plugin Extended Depth of Field (http://bigwww.epfl.ch/demo/edf/). Counts were done in MetaMorph using a custom-made journal suite incorporating the count nuclei function to segment and count the number of macrophages containing diffuse cyan, cyan specks, and DRAQ7 positive cells. To determine the total cell count, macrophages containing diffuse cyan and cyan specks were added together to account for the entire population of cells.

For experiments examining *Candida* inside of macrophages (phagocytosis index in Fig. 2A and length measurements of hyphal cells in Fig. 5E and S7C), *Candida* and macrophages were co-incubated for 1 h, and unphagocytosed cells washed off thrice with PBS. Cells were fixed with 4% paraformaldehyde for 10 min and then quenched for 10 min with 0.1M Tris-Cl, pH 7.4. After washing with PBS, cells were then treated for 5 min in PBS containing 0.1% Triton X-100. After washing with PBS, cells were stained with 10 μg/ml calcofluor white for 10 min, washed 4 times with PBS and mounted on glass slides with Dako fluorescence mounting medium. Imaging was done using the 40x objective with DIC and DAPI filters. Fiji's freehand tool was used to measure cell lengths as described in the Microscopy section.

The colony forming units of *C. albicans* following co-incubation with macrophages were determined as previously described (2), with the exception that after extraction from macrophages, *C. albicans* strains were plated on plates without methionine or cysteine, i.e. permissive for growth of the *mmm1* mutant.

***Quantitative RT-PCR***

Total RNA was extracted by the hot acid phenol method. DNase treatment of 10 μg of RNA using TURBO DNase (Ambion) was performed according to the manufacturer's instructions. One μg of DNase treated total RNA was reverse-transcribed using SuperScript III Reverse Transcriptase (Invitrogen) according to the manufacturer's instructions. qPCR reaction was done on LightCycler 480 (Roche) using the FastStart Universal SYBR Green Master Rox (Roche) reagent mix. Data was analysed with the LinReg software (3, 4). Primers are listed in Table S3.

***Phospholipid analysis***

For experiments involving the conditional ERMES mutants, 5 mL cultures were grown in permissive conditions overnight at 30 °C. At time point 0, cultures were diluted to $OD_{600}$=0.3 in 50 mL permissive or repressive (with or without 2.5 mM methionine and 0.5 mM cysteine) media and grown for 15 h. For experiments involving ERMES deletion strains, 10 mL cultures were grown in YPD for 2 days to account for the very slow growth of the mutant strains. For each sample, a mass of ≈220mg of cells was collected by centrifugation and washed two times with $dH_2O$, followed by lipid extraction using a slightly modified Folch procedure (5, 6). In brief, cells were resuspended in 330 µL of methanol and disrupted by vortexing with 100 µL of glass beads for 10 min. After a pulse centrifugation at high speed, absorbance readings at 280 nm were taken of the supernatant, to approximate the amount of protein in each extract. Subsequently, 660 µL of chloroform was added and samples were vortexed and centrifuged for 10 min at 10000 x g to remove insoluble material. The organic supernatant was recovered and washed once with 100 µL of 0.9 % NaCl and the lower chloroform phase was dried under a stream of nitrogen. Lipids were normalized according to protein levels, resuspending 50 µL of chloroform:ethanol (30:35) per 2.8 mg protein in the starting extract, and 2 µL of each lipid sample was separated by thin layer chromatography (TLC) on Silica 60 plates (Millipore cat # F254S GL, 10x10 cm) in chloroform/ethanol/water/triethylamine (30:35:7:35). Dried TLC plates were then stained with 470 mM $CuSO_4$ in 8% o-phosphoric acid and subsequently dried and incubated for 10 min at 180 °C. Standards were used to identify cardiolipin (CL), phosphatidylethanolamine (PE), phosphatidylcholine (PC), and phosphatidylserine (PS), and were from Avanti Polar Lipids, CL (#710335), PE (#850757), PC (#850457), and PS (#840034). Lipids were quantified using the

Toolbox module of ImageQuant 1D version 7.0, and background signals were subtracted using the local median method performed by the software.

***Analysis of 1,3 β-glucan by flow cytometry***

Surface exposed 1, 3 β-glucan on hyphal cells was stained and quantified as described in (2). Briefly, hyphal cells were stained with the mouse anti-1, 3 β-glucan antibody (Biosupplies Australia) by incubating for 30 min at room temperature, followed by washing with PBS and incubation with AlexaFluor 488-labelled goat anti-mouse IgG (Invitrogen) for a further 30 min at room temperature. After washing with PBS, stained hyphae were analyzed by flow cytometry (FACSCalibur, BD Biosciences) using the FACSDiva 5.0 software (BD Biosciences). The dot plots and different gates were generated in Weasel V3 software (WEHI, Australia).

## REFERENCES:


1. **Croker, B. A., J. A. O'Donnell, C. J. Nowell, D. Metcalf, G. Dewson, K. J. Campbell, K. L. Rogers, Y. Hu, G. K. Smyth, J. G. Zhang, M. White, K. Lackovic, L. H. Cengia, L. A. O'Reilly, P. Bouillet, S. Cory, A. Strasser, and A. W. Roberts.** 2011. Fas-mediated neutrophil apoptosis is accelerated by Bid, Bak, and Bax and inhibited by Bcl-2 and Mcl-1. Proceedings of the National Academy of Sciences of the United States of America **108:**13135-13140.
2. **Uwamahoro, N., J. Verma-Gaur, H. H. Shen, Y. Qu, R. Lewis, J. Lu, K. Bambery, S. L. Masters, J. E. Vince, T. Naderer, and A. Traven.** 2014. The pathogen Candida albicans hijacks pyroptosis for escape from macrophages. mBio **5:**e00003-00014.
3. **Ramakers, C., J. M. Ruijter, R. H. Deprez, and A. F. Moorman.** 2003. Assumption-free analysis of quantitative real-time polymerase chain reaction (PCR) data. Neuroscience letters **339:**62-66.
4. **Ruijter, J. M., C. Ramakers, W. M. Hoogaars, Y. Karlen, O. Bakker, M. J. van den Hoff, and A. F. Moorman.** 2009. Amplification efficiency: linking baseline and bias in the analysis of quantitative PCR data. Nucleic acids research **37:**e45.
5. **Folch, J., M. Lees, and G. H. Sloane Stanley.** 1957. A simple method for the isolation and purification of total lipides from animal tissues. The Journal of biological chemistry **226:**497-509.
6. **Chen, Y. L., A. E. Montedonico, S. Kauffman, J. R. Dunlap, F. M. Menn, and T. B. Reynolds.** 2010. Phosphatidylserine synthase and phosphatidylserine decarboxylase are essential for cell wall integrity and virulence in Candida albicans. Molecular microbiology **75:**1112-1132.
