# Supplementary material for: The Endoplasmic Reticulum-Mitochondrion Tether ERMES Orchestrates Fungal Immune Evasion, Illuminating Inflammasome Responses to Hyphal Signals
Source: mSphere. 2016 May 25;1(3):e00074-16. doi: 10.1128/mSphere.00074-16 (PMC4888881; doi:10.1128/mSphere.00074-16)
Supplement: Tables S1 to S3 [file sph003162089st3.pdf]

Table S1

**Strain**	**Genotype**	**Traven collection reference**
BWP17	*ura3Δ::λimm434/ura3Δ::λimm434*<br>*arg4Δ::hisG/arg4Δ::hisG*<br>*his1Δ::hisG/his1Δ::hisG*	YCAT15
DAY185	*ura3Δ::λimm434/ura3Δ::λimm434*<br>*arg4Δ::hisG/ARG4::URA3::arg4Δ::hisG*<br>*his1Δ::hisG/his1Δ::hisG::pHIS1*	YCAT504
DAY286	*ura3Δ::λimm434/ura3Δ::λimm434*<br>*arg4Δ::hisG/ARG4::URA3::arg4Δ::hisG*<br>*his1Δ::hisG/his::hisG*	YCAT14
mdm10	*ura3Δ::λimm434/ura3Δ::λimm434*<br>*arg4Δ::hisG/arg4Δ::hisG*<br>*his1Δ::hisG/his1Δ::hisG::pHIS1*<br>*mdm10Δ::ARG4/URA3-*P_{MET3}*-MDM10*	YCAT597
mmm1	*ura3Δ::λimm434/ura3Δ::λimm434*<br>*arg4Δ::hisG/arg4Δ::hisG*<br>*his1Δ::hisG/his1Δ::hisG::pHIS1*<br>*mmm1Δ::ARG4/URA3-*P_{MET3}*-MMM1*	YCAT595
mmm1+1	*ura3Δ::λimm434/ura3Δ::λimm434*<br>*arg4Δ::hisG/arg4Δ::hisG*<br>*his1Δ::hisG/hisΔ::hisG::pHIS1-MMM1*<br>*mmm1Δ::ARG4/URA3-*P_{MET3}*-MMM1*	YCAT697
mdm12ΔΔ	*ura3Δ::λimm434/ura3Δ::λimm434*	YCAT735

	arg4Δ::hisG/arg4Δ::hisG<br>*his1Δ::hisG/hisΔ::hisG::pHIS1*<br>*mdm12Δ::URA3/mdm12Δ::ARG4*<br>*OR*<br>*ura3Δ::λimm434/ura3Δ::λimm434*<br>*arg4Δ::hisG/arg4Δ::hisG*<br>*his1Δ::hisG /hisΔ::hisG*<br>*mdm12Δ::URA3/mdm12Δ::ARG4*	YCAT711
mdm12ΔΔ+12	*ura3Δ::λimm434/ura3Δ::λimm434*<br>*arg4Δ::hisG/arg4Δ::hisG*<br>*his1Δ::hisG/hisΔ::hisG::pHIS-MDM12*<br>*mdm12Δ::URA3/mdm12Δ::ARG4*	YCAT741
mmm1ΔΔ	*ura3Δ::λimm434/ura3Δ::λimm434*<br>*arg4Δ::hisG/arg4Δ::hisG*<br>*his1Δ::hisG/hisΔ::hisG::pHIS1*<br>*mmm1Δ::URA3/mmm1Δ::ARG4*<br>*OR*<br>*ura3Δ::λimm434/ura3Δ::λimm434*<br>*arg4Δ::hisG/arg4Δ::hisG*<br>*his1Δ::hisG/hisΔ::hisG*<br>*mmm1Δ::URA3/mmm1Δ::ARG4*	YCAT734<br><br>YCAT705
mmm1ΔΔ+1	*ura3Δ::λimm434/ura3Δ::λimm434*<br>*arg4Δ::hisG/arg4Δ::hisG*<br>*his1Δ::hisG/hisΔ::hisG::pHIS1-MMM1*<br>*mmm1Δ::URA3/mmm1Δ::ARG4*	YCAT739
mdm10ΔΔ	*ura3Δ::λimm434/ura3Δ::λimm434*	YCAT726

	arg4Δ::hisG/arg4Δ::hisG<br>*his1Δ::hisG/hisΔ::hisG*<br>*mdm10Δ::URA3/mdm10Δ::ARG4*	
mdm34ΔΔ	*ura3Δ::λimm434/ura3Δ::λimm434*<br>*arg4Δ::hisG/arg4Δ::hisG*<br>*his1Δ::hisG/hisΔ::hisG::pHIS1*<br>*mdm34Δ::URA3/mdm34Δ::ARG4*<br>*OR*<br>*ura3Δ::λimm434/ura3Δ::λimm434*<br>*arg4Δ::hisG/arg4Δ::hisG*<br>*his1Δ::hisG/hisΔ::hisG*<br>*mdm34Δ::URA3/mdm34Δ::ARG4*	YCAT737<br><br><br><br><br>YCAT720
mdm34ΔΔ+34	*ura3Δ::λimm434/ura3Δ::λimm434*<br>*arg4Δ::hisG/arg4Δ::hisG*<br>*his1Δ::hisG/hisΔ::hisG::pHIS1-MDM34*<br>*mdm34Δ::URA3/mdm34Δ::ARG4*	YCAT747

Table S2.

Label	Primer sequence
MMM1 KO F'	ATAGTGCCAGAGATTTCACAGTGGGGGGGAGAAAAACTTCCAAAAAATATCTTCTCTAGTACCACATGAAGATATTACAGATTGCAAATCAAGTTGATCATTTCCCAGTCACGACGTTGTAAAAC
MMM1 KO R'	CTGGATTTGTACTACTTCACCACTACATCAGTTGAAGGTCTTAAACTAAAGAAAGATATACATCTCACTTCATTTAATAAATGTACATATACACTGCAATGTGGAATTGTGAGCGGATA
MMM1 MET R'	TCAATTGTTCAAGTAAAGAGTCATGGATTTGATGACCTAGTTCTCTTGCCTCAACTATTTTTGTTGTCGTTGCTGTTGTTTCAATTAAATCCTGTGACATGTTTTCTGGGGAGGGTATTTACTTT
MDM10 KO F'	CAATTAGCCTTTTTAATTTGTTGTTTAGATACTTACTTTTCCCCCTTTTTTAATTTAATTCAATTCAACTGGTTTCTTTTTTTCGGGGTTAGTTTTTCAATTTCCCAGTCACGACGTT
MDM10 KO R'	GTAATATTTAATGCTACACTTACTCTATCTTCATCTCTATCTATCTATCAAAGGTATGGTTCTATATTATTTAACTAACCTATCTAGCAACTTACTTCTTGTGGAATTGTGAGCGGATA
MDM10 MET R'	AAGCTTGAGATGTGGCAGTAATATTAGAATATATATTGTCTTCATTCCAATTAGTTGATTTATAAAAACATTTCTGTAAGTATTCCATATATGTATACATGTTTTCTGGGGAGGGTATTTACTTT
MDM12 KO F'	CGACTTATTAAATTTTCATAAGTAAAGTCATTTTGAAGTGTATAACGTTCTCTCTTTACCAACCGATGACAACACCCAGTTAACGCAAGCATCATTATAATTTCCCAGTCACGACGTTGTAAAAC
MDM12 KO R'	GCTATTACTATGTAATAATTTGATTTCTCAACCATATTGACCTGATATCGACTTTCTTTGTAGCAATTCATTTATGTCCATTTAGCACATTGTGGTGATTAGTGGAATTGTGAGCGGATA
MDM12 MET R'	GGAGTGAGATATTTTTGAATTGCTGATCCAAAAACTCTTTTATCGA

	TTGATTGATCGTGTCATCAATTGTTAATTGATTCCAATTAATATCAA ATGACATGTTTTCTGGGGAGGGTATTTACTTT
MDM34 KO F'	ACCTTCTCCCCTTCCCCCTTATAACTTCCAATTCTAACGGGTTATTC GTACTTTGGAAACCAGCTAATCCTATAGCTTTTTGCACACTTTTTTT ATAAACTTTCCCAGTCACGACGTT
MDM34 KO R'	AAATAAAACAATCCAACATAGTATTTGTCATCTCTCATACTCTTTAT GTAATAGATTTACGTTTACAATAAAAGAAACTCAAAATTCCATAAC CTTCATGGTGGAATTGTGAGCGGATA
MMM1 CHECK F'	ACCGACAAGATTTCAGTGTA
MMM1 CHECK R'	AAATAACTCATGACTACGCC
MDM10 CHECK F'	ACAAACACCAAGAAGTTAAA
MDM10 CHECK R'	GAAGCCAGATAATCTGTAGT
MDM12 CHECK F'	ACATGTTACTGACTCAGCTA
MDM12 CHECK R'	GGCTATTGGCGTGTTAGGTG
MDM34 CHECK F'	AGACTTATAACACACACCAA
MDM34 CHECK R'	TGTCACTAACAGACATTTAC
ARG4 CHECK F'	GGAATTGATCAATTATCTTTTGAAC
ARG4 CHECK R'	AAGTACACGACCCACAGTTA
URA3 CHECK F'	ATGGCACTACAGCAACTTTC
URA3 CHECK R'	AACGCGTTGGATGCATAGCT

Table S3.

Gene	Primer sequence
[1]SCR1 F'	TTTAGCATAACCACTGGAGGGAAG
[1]SCR1 R'	GAGTTGCAACACTAGATACCGCACT
[2]RDN5 F’	CATATCTAGCAGAAAGCACCGTTC
[2]RDN5 R’	ACAATAGTTTCGCGTATGGTCTC
[2]RDN25 F’	GAGAGGAACCGTTCATTCAGATAAT
[2]RDN25 R’	CTATGGTCCAGCGACTAAAAAGTCT
[2]MMM1 F'	GACTACCTTCATTATGGCCTAGAACT
[2]MMM1 R'	TAGCTGCACTCGTACCGTTTAC
[2]MDM10 F'	ACCCTGCTGTGATTCCCAAA
[2]MDM10 R'	TGCTGCTGATAATCCAGGAC
[2]HWP1 F’	AATCCTCCTCAACCTGATCAGCCTG
[2]HWP1 R’	AGCTGGAGTTGTTGGCTTTTCTGGA
[2]ECE1 F’	TGCCGTCGTCAGATTGCCAGA
[2]ECE1 R’	AGGCCAACATCTGGAACGCCA
[1]PHR1 F’	CTAATTTGCCACCAACTCCA
[1]PHR1 R’	TCGTCAGCAACAACACATTC
[3]SAP5 F’	AGAATTTCCCGTCGATGAGACTGGT
[3]SAP5 R’	CAAATTTTGGGAAGTGCGGGAAGA
[3]SAP6 F’	CCCGTTTTGAAATTAAATATGCTGATGG
[3]SAP6 R’	GTCGTAAGGAGTTCTGGTAGCTTCG

[1]Reference: Verma-Gaur, J., Y. Qu, P. F. Harrison, T. L. Lo, T. Quenault, M. J. Dagley M., Bellousoff, D. R. Powell, T. H. Beilharz, and A. Traven. 2015. Integration of Posttranscriptional Gene Networks into Metabolic Adaptation and Biofilm Maturation in Candida albicans. PLoS genetics **11:**e1005590.
[2]Reference: This study
[3]Reference: Naglik, J. R., C. A. Rodgers, P. J. Shirlaw, J. L. Dobbie, L. L. Fernandes-Naglik, D. Greenspan, N. Agabian, and S. J. Challacombe. 2003. Differential expression of Candida albicans secreted aspartyl proteinase and phospholipase B genes in humans

correlates with active oral and vaginal infections. The Journal of infectious diseases 188:469-479.
